# Seek and ye shall find: new species and new records of *Microporella* (Bryozoa, Cheilostomatida) in the Mediterranean

**DOI:** 10.3897/zookeys.1053.65324

**Published:** 2021-08-02

**Authors:** Emanuela Di Martino, Antonietta Rosso

**Affiliations:** 1 Natural History Museum, University of Oslo – Blindern, P.O. Box 1172, Oslo 0318, Norway University of Oslo Oslo Norway; 2 Dipartimento di Scienze Biologiche, Geologiche e Ambientali, Università di Catania – Corso Italia 57, 95129, Catania, Italy Università di Catania Catania Italy; 3 CoNISMa – Consorzio Interuniversitario per le Scienze del Mare – Piazzale Flaminio, 9, 00196, Roma, Italy Consorzio Interuniversitario per le Scienze del Mare Rome Italy

**Keywords:** Biodiversity, cryptic species, detritic bottoms, *
Diporula
*, Infralittoral Algae Biocoenosis, Ionian Sea, marine protected areas, submarine caves

## Abstract

The Mediterranean specimens of the genus *Microporella* collected from shallow water habitats during several surveys and cruises undertaken mostly off the Italian coast are revised. As a result of the disentanglement of the *M.ciliata* complex and the examination of new material, three new species, *M.bicollaris***sp. nov.**, *M.ichnusae***sp. nov.**, and *M.pachyspina***sp. nov.**, are described from submarine caves or associated with seagrasses and algae. An additional species *Microporella* sp. A, distinct by its finely reticulate ascopore, is described but left in open nomenclature owing to the limitations of a single infertile fragment. After examination of all available material, based on their identical zooidal morphology, the genus *Diporula* is regarded as junior synonym of *Microporella* and the combination *Microporellaverrucosa* is resurrected as first suggested by Neviani in 1896. *Fenestrulinajoannae* is also reassigned to *Microporella*. The availability of a large number of colonies of the above-mentioned and other species already well known from the area (i.e., *M.appendiculata*, *M.ciliata*, and *M.modesta*), allowed the assessment of their high intraspecific variability as well as the observation, for the first time, of some morphological characters including ancestrulae, early astogeny, and kenozooids. Finally, *M.modesta*, in spite of *M.ciliata* as defined by the neotype selected by Kukliński & Taylor in 2008, appears to be the commonest species in the basin.

## Introduction

The genus *Microporella* Hincks, 1877 is one of the most distinctive bryozoan genera (Taylor and Mawatari 2005), both cosmopolitan and species-rich, currently including 34 fossil and 115 Recent species (Bock 2020). These numbers are constantly increasing, with new species of *Microporella* continuously being described worldwide, both fossil (e.g., Di Martino et al. 2017, 2019, 2020a) and Recent (e.g., Almeida et al. 2017; Figuerola et al. 2018; Dick et al. 2020; Di Martino et al. 2020a; Ramalho et al. 2020).

In the Mediterranean, *Microporella* is represented to date by nine species (Rosso and Di Martino 2016; Di Martino et al. 2020a), and knowledge of the diversity of the genus in the basin has been stable during the last decade. The last thorough revision was by Harmelin et al. (2011), but was limited to the south-eastern sectors of the Mediterranean along the coast of Lebanon. This work increased the number of *Microporella* species recorded from the Mediterranean, but also showed how the stock of *Microporella* species found in this area was composed only of tropical alien species.

Here, we examine large volumes of material, collected along the Italian coast in several shallow water habitats, to assess the diversity of the genus in these sectors of the Mediterranean, the morphological intraspecific variability of the *Microporella* species found, as well as their distribution and commonness. We also describe three new species and, for those previously known, illustrate for the first time some key morphological features. An additional species is described and left unnamed. Although some of its morphological features are distinct from other congeners (e.g., ascopore), the lack of some diagnostic characters, such as ovicells, and the scarcity of material available prevents the description of a new species.

## Materials and methods

**Figure 1.**
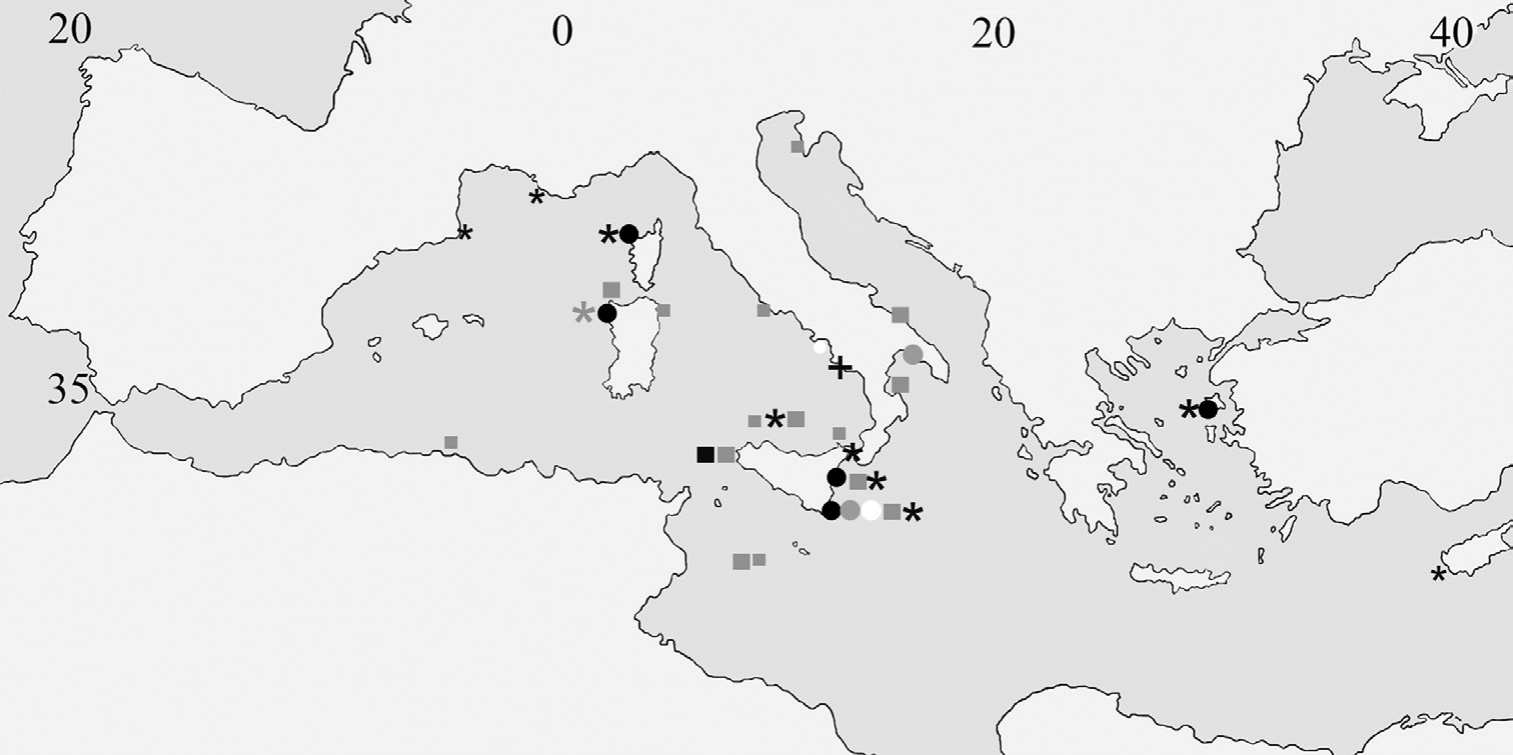
Geographical distribution of the Mediterranean species of *Microporella* studied in this paper, either based on examined material (larger symbols) or on data from the literature (Kukliński and Taylor 2008 for *M.ciliata*; Hayward and McKinney 2002; Chimenz Gusso et al. 2014 and Di Martino et al. 2020a for *M.modesta*; André et al. 2014 and Achilleos et al. 2020 for *M.verrucosa*) that include SEM images (smaller symbols). Black circles: *M.appendiculata*; grey circles: *M.bicollaris* sp. nov.; white circles: *M.ciliata*; grey asterisk: *M.ichnusae* sp. nov.; grey squares: *M.modesta*; black squares: *M.pachyspina* sp. nov.; black cross: *Microporella* sp. A; black asterisks: *M.verrucosa*.

This study is based on material collected during the last 40 years in several biodiversity surveys carried out under the umbrella of different projects summarised in Table 1, which also provides collection data for each described species. The studied area with the distribution of species is shown in Fig. 1. Further details for some cruises can be found in the following papers: PS/81: Piattaforma Siciliana cruise (Rosso 1989; 1996a; 1996b); N/96: Noto cruise (Cantone 1997); Mazzere and Granchi caves, Plemmirio Marine Protected Area (MPA) (Rosso et al. 2013a); LCT: Gulf of Catania cruise (Poluzzi and Rosso 1988; Rosso 1989); CI: Ciclopi 2000 cruise (Rosso 2001); SM: Ciclopi Islands MPA (Rosso et al. 2019a); AM: Amendolara Bank cruise (Di Geronimo et al. 1998); CL: Calvì cruise (Rosso 1989); Bari Canyon (D’Onghia et al. 2015); Lesvos Island, Agios Vasilios cave (Rosso et al. 2019b).

Scanning electron microscopy (SEM) was conducted on uncoated specimens using a TESCAN VEGA 2 LMU in backscattered-electron/low-vacuum mode at the Microscopical Laboratory of the University of Catania. Measurements were taken from SEM micrographs using the image processing program ImageJ (available from https://imagej.nih.gov/) and are given in the species descriptions and/or in the remarks as ranges and, in parentheses, mean ± standard deviation plus the number of zooids measured (N). Abbreviations for the measurements are:

**AvL** avicularium length;

**AvW** avicularium width;

**OL** orifice length;

**OW** orifice width;

**OvL** ovicell length;

**OvW** ovicell width;

**ZL** autozooid length;

**ZW** autozooid width.

Type material of the new species and figured specimens form part of the Rosso Collection deposited at the Museum of Palaeontology of the University of Catania (**PMC**) under the catalogue numbers reported for each species.

Note that we follow Harmelin et al. (2011) for the authorships of *Microporellacoronata* (Audouin & Savigny, 1826) and *M.genisii* (Audouin & Savigny, 1826).

## Taxonomic treatments

### Phylum Bryozoa Ehrenberg, 1831

#### Class Gymnolaemata Allman, 1856

**Order Cheilostomatida Busk, 1852**

**Superfamily Schizoporelloidea Jullien, 1883**

**Family Microporellidae Hincks, 1879**

##### Genus Microporella

Taxon classificationAnimaliaCheilostomatidaMicroporellidae

Hincks, 1877

160920B2-45B7-5C85-A507-4BA0A8D1E0A3

###### Type species.

*Escharaciliata* Pallas, 1766

##### Microporella appendiculata

Taxon classificationAnimaliaCheilostomatidaMicroporellidae

(Heller, 1867)

30564729-1C25-5456-859F-22FC3B5FDE44

Fig. 2

Lepralia appendiculata Heller, 1867: 107, pl. 2, fig. 8.
?Microporellacoronata (Audouin & Savigny, 1826): Gautier 1962: 173.
Microporella coronata (Audouin & Savigny): Zabala 1986: 513, fig. 180.
Microporella marsupiata (Busk, 1860): Zabala 1986: 514, fig. 181, pl. 15D.
Microporella pseudomarsupiata Arístegui, 1984: 325, pl. 24, fig. 6; Zabala and Maluquer 1988: 141, fig. 335, pl. 19C; Di Geronimo et al. 1993a: table 1; Di Geronimo et al. 1997: table 2; Chimenz and Faraglia 1995: 40, table 1, pl. 2C; Rosso 1996a: table 2.
Microporella appendiculata (Heller): Hayward and Ryland 1999: 294, figs 134A, B, 135 (cum syn.); Chimenz Gusso et al. 2014: 187, fig. 100a–e.

###### Examined material.

Italy • 2 living colonies; Ionian Sea, E Sicily, Ciclopi Island MPA; samples Ciclopi 2000 4E and 14G; 37°32'2﻿8"–37°34'30"N, 15°8'59"–15°11'1"E; 52 and 90 m; 16 Jul. 2000; A. Rosso leg.; dredging; DC and DL Biocoenoses; PMC Rosso-Collection I. H. B.84a. Italy • 27 living and 10 dead colonies/fragments; Ionian Sea, SE Sicily, Gulf of Noto; 36°41'45"–36°57'48"N, 15°8'35"–15°20'0"E; PS/81 cruise; samples CR1, 9B and 10C; 45, 44 and 60 m; Jul. 1981; I. Di Geronimo leg.; dredging; DC Biocoenoses; and 3 living colonies; Noto 1996 cruise; samples 6C and 9E; 45–50 m; 1996; E. Mollica leg.; dredging; VTC and DC Biocoenoses; PMC Rosso-collection I. H. B84c. Italy • 5 living colonies; Iberian-Provençal Basin, NW Sardinia, Capo Caccia-Punta Giglio MPA; samples Bisbe 1, Bisbe 2 and Falco 1; 40°35'40"N, 8°11'39"E; 7–8 m; Jun. 2009; V. Di Martino leg.; submarine cave; scuba diving; PMC Rosso-Collection I. H. B.84b. France • 11 dead colonies; Iberian-Provençal Basin, Corsica, off Calvì; sample CL 74; 42°47'31"N, 9°8'10"E; 150–110 m; G. Fredj leg.; dredging; DL Biocoenosis; PMC Rosso-collection Fr. H. B84d. Greece • 1 dead colony; NE Aegean Sea, Lesvos Island, Agios Vasilios cave; sample AV1; 38°58'9"N, 26°32'28"E; ca. 30 m, V. Gerovasileiou leg.; submarine cave; scuba diving; PMC Rosso-collection Gr. H. B84e.

**Figure 2.**
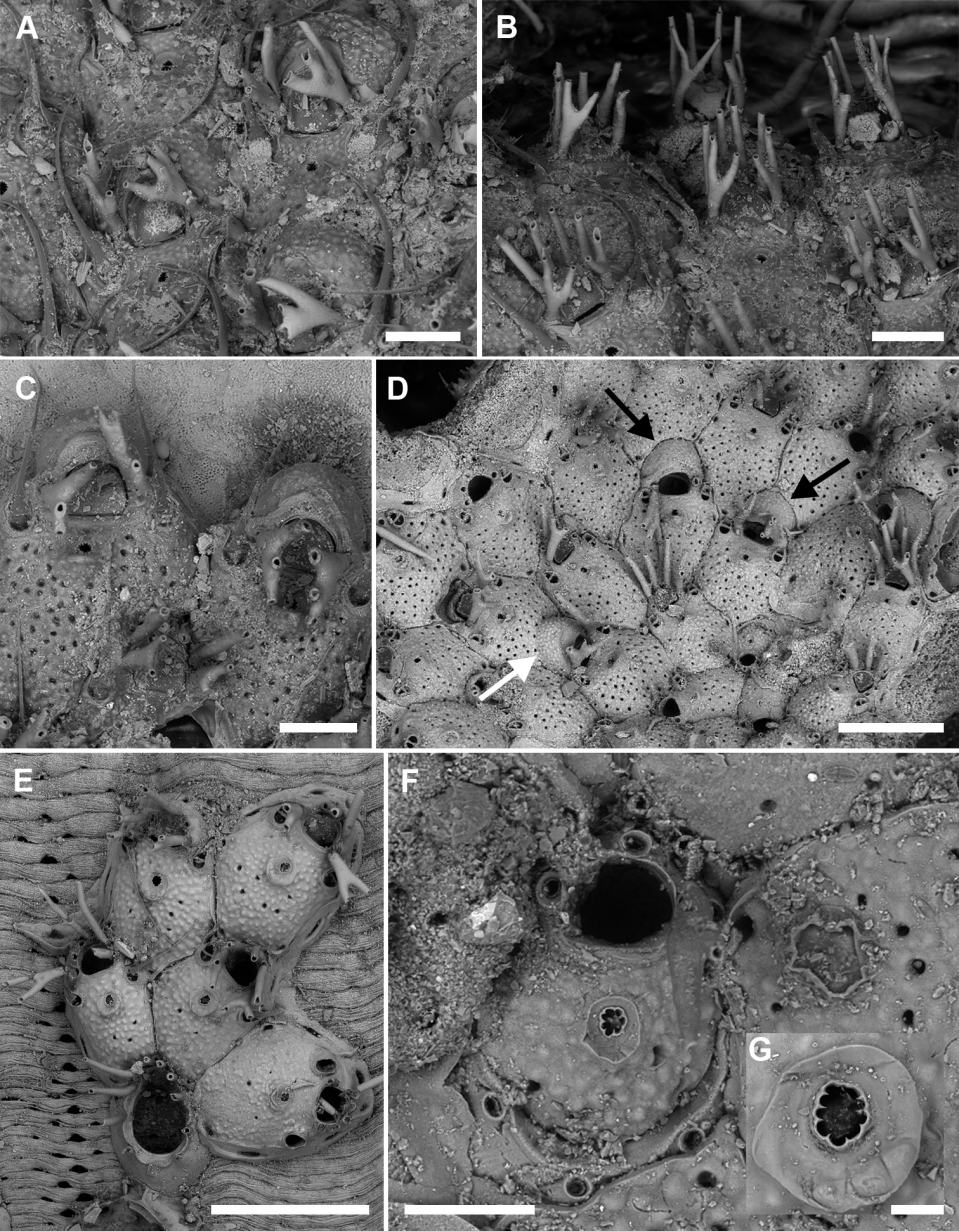
*Microporellaappendiculata* (Heller, 1867) from the Iberian-Provençal Basin **A, B** Bisbe cave **E** Falco Cave, PMC Rosso-Collection I. H. B.84b **C, D, F, G** Ionian Sea, Ciclopi 2000, PMC Rosso-Collection I. H. B.84a **A** unbleached autozooids with trifurcate proximal oral spines and long, setiform avicularian mandibles **B** unbleached autozooids with bifurcate proximal oral spines. Note the vertical arrangement of the unbranched distal spines forming a sort of fence around the orifice **C** two kenozooidal ovicells developed on marginal autozooids **D** colony portion showing the co-occurrence of kenozooidal ovicells (black arrows) and ovicells formed by the distal autozooid (white arrow). Note the occurrence of bifurcate and trifurcate proximal spines **E** young colony showing the tatiform ancestrula and first budded autozooids with bifurcate proximal spines **F** ancestrula regenerated as a miniaturised autozooid **G** close-up of the ascopore. Scale bars: 200 µm (**A–D**); 500 µm (**E**); 100 µm (**F**); 30 µm (**G**).

###### Description.

Colony encrusting multiserial, unilaminar, forming subcircular patches; interzooidal communications typically via two proximolateral, two distolateral and three distal pore-chamber windows, 48–122 (71±25, N = 10) × 16–26 μm (20±3, N = 10) along lateral walls.

Autozooids polygonal, 529–742 (644±66, N = 14) × 347–582 (458±66, N = 14) μm (mean L/W = 1.41), distinct, the boundaries marked by narrow grooves between the slightly raised vertical walls (Fig. 2D, E). Frontal shield flat to slightly convex, coarsely, densely and evenly granular; 5–8 marginal areolae only occasionally distinguishable from pseudopores; pseudopores circular to elliptical (6–16 μm in diameter), numbering 30–42 (fewer in periancestrular zooids), placed in the proximal half of the zooid (Fig. 2E); area between orifice and ascopore imperforate.

Primary orifice transversely D-shaped, 100–110 (105±7, N = 2) × 129–141 μm (135±8, N = 2) (mean OL/OW = 0.78; mean ZL/OL = 6.14); hinge-line straight or concave, smooth, without condyles and denticles. Five, occasionally six, articulated oral spines, 170–310 μm long (diameter of the base 25–42 μm), the proximalmost pair bi- to trifurcated, the tips sometimes curved towards the centre of the orifice (Fig. 2A, B); joints brown.

Ascopore field an area of smooth, gymnocystal calcification, placed 50–80 μm below the orifice, transversely elliptical, 48–122 × 61–110 μm, narrow distally, more extensive proximally and developing a prominent, pointed mucro not concealing the ascopore; ascopore subcircular, 22–43 μm in diameter, with a dozen of radial spines (Fig. 2F, G).

Avicularia paired, 72–170 (103±29, N = 37) × 46–103 (67±13, N = 37) μm (mean AvL/AvW = 1.53), located distolaterally, the complete crossbar at the same level of the orifice hinge-line (Fig. 2D, E); rostrum short, arched or truncated distally and open-ended, distally directed. Mandible brown, 387–659 μm long, setiform, typically curved, lying on the distal zooid (Fig. 2A).

Ovicell subglobular and semi-immersed, 143–235 (195±29, N = 15) × 262–378 (329±38, N = 15) μm (mean OvL/OvW = 0.59), non-personate, not obscuring the proximal part of the orifice, closed by the operculum during brooding (Fig. 2A, C, D), in the same colony either kenozooidal (Fig. 2C, D black arrows) or produced by the distal autozooid (Fig. 2D white arrow); surface finely granular, imperforate; a proximal rim of gymnocystal calcification occupying about one-third of the ooecium length, forming a raised visor-like rim; proximalmost pair of spines (branched) visible in ovicellate zooids.

Ancestrula tatiform (Fig. 2E), oval (424 × 324 μm), gymnocyst more extensive proximally than laterally, about 150 μm wide; opesia pyriform (270 × 180 μm), surrounded by a smooth, flared cryptocyst with the undulate marginal rim indented by ten gymnocystal spines (six distal, two median, three proximal). Ancestrula budding two distolateral autozooids, often regenerated as a small autozooid lacking avicularia (Fig. 2F).

###### Remarks.

Originally described from the Adriatic by Heller (1867), *Microporellaappendiculata* has often been recorded as *Microporellamarsupiata* Busk, 1860 or as *M.pseudomarsupiata* Arístegui, 1984 (see Hayward and Ryland 1999 and references therein; Rosso et al. 2010; Chimenz Gusso et al. 2014). With the exception of Hayward and Ryland (1999) and recent online material such as the catalogue of the Museu de Ciènces Natural de Barcelona (https://zoologiaenlinia.museuciencies.cat/detall/zoologia_general/N1064271/) and the website of the program DORIS (https://doris.ffessm.fr/Especes/Microporella-pseudomarsupiata-Microporelle-ciliee-noire-1695), a comprehensive description and illustration of this species is absent in the literature.

*Microporellaflabelligera* Levinsen, 1909 described from the vicinity of Siracusa, at depths (28–46 m) comparable to those of the PS/81 sites in the Gulf of Noto, is likely to be conspecific with *M.appendiculata* based on the original drawings (Levinsen 1909: 331, pl. 24 cited as 23 in the text, fig. 6A–C). However, a formal synonymy requires the examination of the type specimen.

*Microporellaappendiculata* differs from other Mediterranean congeners in having paired avicularia, a character shared only with *M.coronata* (Audouin & Savigny, 1826). However, in *M.coronata* the avicularia are located proximally to the ascopore, the ovicell is personate, the oral spines are greater in number (6–8) and unbranched.

Here, we document the regeneration of the ancestrula as an autozooid for the first time (Fig. 2F). Trifurcated proximal spines have already been figured on some zooids, though not described as such by Zabala (1986: fig. 181B, as *M.marsupiata*) and by Chimenz and Faraglia (1995: pl. 2C, as *M.pseudomarsupiata*). Gautier (1962) also recorded autozooids with up to seven oral spines.

Size differences were observed between specimens from Sicily and those from Sardinia, with Sardinian colonies showing longer autozooids (Sicily: mean 604±56 × 458±71 μm, N = 8, L/W 1.32; Sardinia: 698±30 × 458±64 μm, N = 6, L/W 1.53), slender avicularia (Sicily: mean 135±7 × 105±8 μm, N = 25, L/W 1.38; Sardinia: 142±14 × 80±14 μm, N = 12, L/W 1.78), and larger ovicells (Sicily: mean 173±20 × 280±14 μm, N = 5, L/W 0.78; Sardinia: 206±27 × 354±13 μm, N = 10, L/W 0.58), as well as trifurcated proximalmost spines.

###### Distribution and ecology.

In the Mediterranean, *M.appendiculata* has been reported from several localities and usually in shelf habitats, associated with coarse detritic bottoms, often encrusting shells and calcareous algae (Gautier 1962; Rosso 1996a, b; Madurell et al. 2013; Chimenz Gusso et al. 2014), as well as from shadowed microhabitats associated with *Cystoseira* s. l. communities (e.g., Campisi 1973; Rosso et al. 2019a). It also occurs in submarine caves of NW Sardinia (Fraschetti et al. 2010) and near Monaco (J.-G. Harmelin, pers. comm., May 2021) in the Iberian-Provençal basin, at the transition between coralligenous and semi-dark cave habitats. *Microporellaappendiculata* has been also collected in dark sectors of Agios Vasilios cave in Lesvos, Greece (Rosso et al. 2019b) and (as *M.umbracula*) in the Mitigliano cave in the Sorrento peninsula, Italy (Balduzzi et al. 1989; Balduzzi and Rosso 2003).

##### Microporella bicollaris sp. nov.

Taxon classificationAnimaliaCheilostomatidaMicroporellidae

E820C8E6-652A-5CD2-8446-FC68D3407541

http://zoobank.org/1C5E0C9C-D08C-48B5-8E7D-8DFB6D6F480D

Fig. 3

Microporella sp. C Rosso et al. 2013a: table 17.1; Rosso et al. 2013b: table 1.

###### Type material.

***Holotype***: Italy • The largest of 2 living colonies on the basal part of a thallus of *Halimedatuna* (Ellis & Solander) Lamoroux, including the ancestrula and several ovicellate zooids; northern Ionian Sea, Gulf of Taranto, Porto Cesareo MPA; sample PCE10; 40°15'54"N, 17°52'38"E; 5–15 m; 2008; A. Sinagra leg.; scuba diving; C Biocoenosis; ***Paratypes***: Italy • 1 dead colony fragment consisting of about a dozen zooids, some fertile; sample PCI10; same details as the holotype; PMC. B29b1. 20.11.2020; 1 dead colony fragment consisting of 9 zooids, 3 of which fertile; Ionian Sea, SE Sicily, Plemmirio MPA, Mazzere submarine cave; sample MZ1 (sediment); 37°00'18"N, 15°18'36"E; 23 m; 14 Sep. 2009; V. Di Martino leg.; scuba diving; C and GSO Biocoenoses; PMC. B29b2. 20.11.2020.

###### Diagnosis.

Colony encrusting, multiserial. Autozooid frontal shield densely pustulose and centrally pseudoporous. Orifice transversely D-shaped; hinge-line smooth with rectangular condyles at corners; five or six oral spines, two visible in ovicellate zooids. Ascopore field circular to elliptical; ascopore opening bean-shaped, with small tongue and radial spines. Avicularium single, located at half zooidal length, directed laterally or slightly disto-laterally; crossbar complete; rostrum lanceolate, channelled. Ovicell produced by the distal zooid, personate with collar enclosing the ascopore and forming a bridge between the orifice and the ascopore, producing two secondary openings.

**Figure 3.**
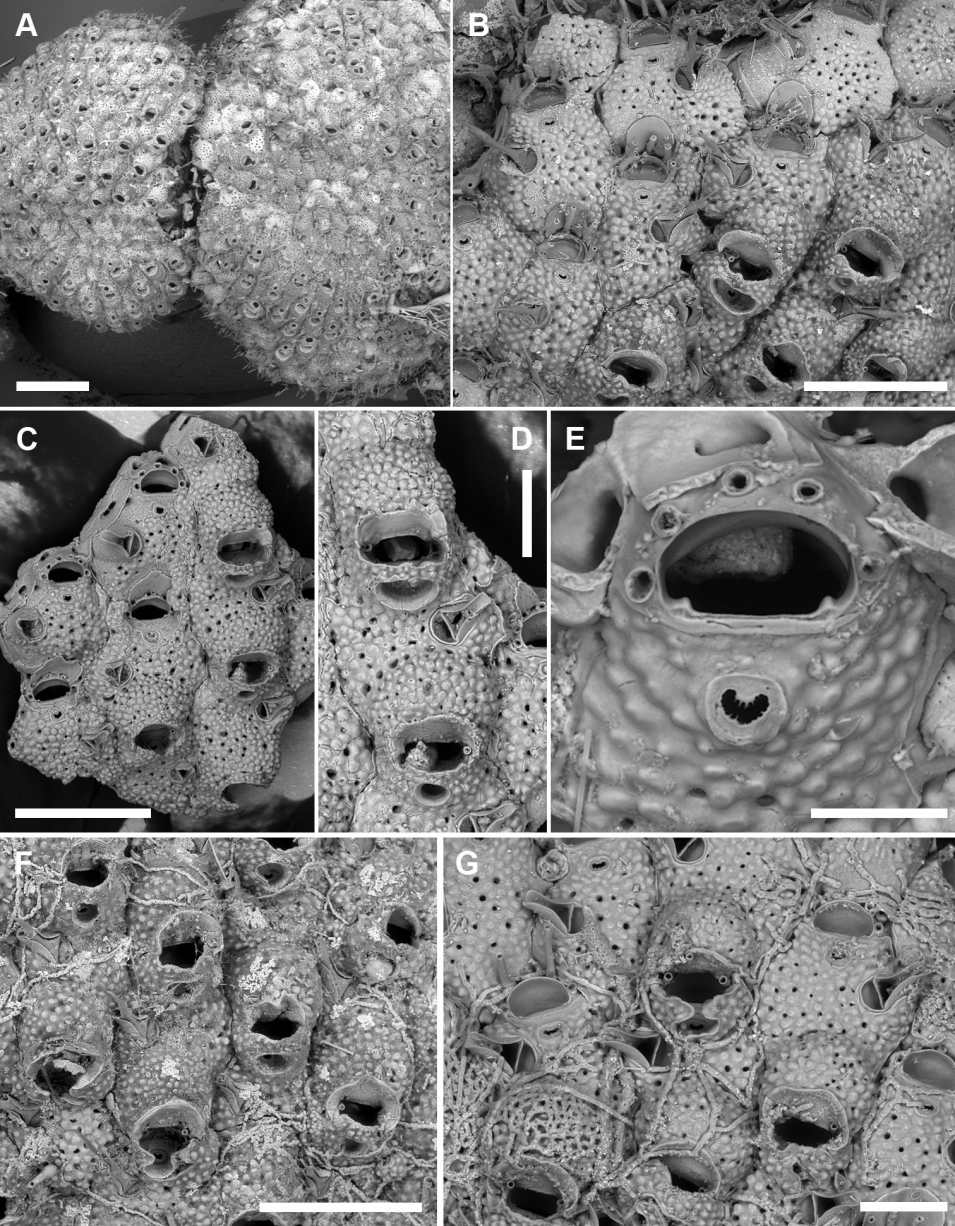
*Microporellabicollaris* sp. nov. from the Ionian Sea **A, B, F** Gulf of Taranto, Porto Cesareo MPA, sample PCE 10 Holotype, PMC. B29a. 20.11.2020 **C, D** SE Sicily, Plemmirio MPA, Mazzere cave, Paratype PMC. B29b2. 20.11.2020 **E, G** Gulf of Taranto, Porto Cesareo MPA, sample PCI 10, Paratype, PMC. B29b1. 20.11.2020 **A** two colonies nearly completely covering subsequent segments of the green alga *Halimedatuna***B** peripheral colony portion, showing the transition from the older autozooids (ovicellate, bottom right) to younger autozooids (non-ovicellate, top left) **C** group of autozooids, one with six oral spine bases (centre left) **D** close-up of two ovicellate zooids with the typical personate ovicell forming a collar bridge between the orifice and the ascopore **E** close-up of the orifice with five spine bases and smooth hinge-line with two rectangular condyles at corners **F** unbleached group of ovicellate autozooids with well-developed collars and avicularian mandibles **G** group of bleached autozooids, some ovicellate, fouled by algae. Note the collar developing from converging lateral lappets, the vizor-like band of gymnocystal calcification leaving visible the proximalmost pair of oral spines. Scale bars: 1 mm (**A**); 500 µm (**B, C, F**); 200 µm (**D, G**); 100 µm (**E**).

###### Description.

Colony encrusting, multiserial, unilaminar (Fig. 3A); interzooidal communications through four elliptical, lateral (two proximo- and two disto-lateral), and two rounded, distal pore chamber windows (38–67 × 16–21 µm).

Autozooids hexagonal, 460–522 (494±31, N = 3) × 411–476 (433±37, N = 3) µm (mean L/W = 1.16), boundaries marked by narrow, sinuous grooves and/or a raised rim. Frontal shield slightly convex, densely and evenly pustulose, with 11–25 circular (diameter 5–20 µm) pseudopores, irregularly distributed centrally; 3–6 marginal areolae, often indistinguishable from pseudopores (Fig. 3C, D).

Orifice transversely D-shaped, 83–95 (89±5, N = 6) × 141–170 (150±11, N = 6) µm (mean OL/OW = 0.60; mean ZL/OL = 5.47), outlined by a thin, raised (relative to the surrounding frontal shield) rim; hinge-line straight, smooth, with a pair of rectangular condyles at corners (Fig. 3E). Oral spines five or six (diameter of the base 18–27 μm), evenly spaced (Fig. 3C, E); proximalmost pair of spines sometimes visible in ovicellate autozooids, embedded between the proximal margin of the ooecium and the personate collar (Fig. 3D, G).

Ascopore field a very narrow, subcircular area of gymnocystal calcification, 35–42 × 46–70 μm, located 35–47 μm below the orifice, at the same level as the orifice but slightly raised relative to the adjacent frontal shield; opening bean-shaped, 32–37 × 9–19 μm, with a small, subcircular tongue projecting from distal edge and tiny radial denticles (Fig. 3E).

Avicularium single, relatively large, 134–190 (165±18, N = 10) × 86–109 (97±9, N = 10) μm (mean AvL/AvW = 1.70), located laterally, on either side, at about half zooidal length (Fig. 3B–D, G); crossbar complete, thin; rostrum long, lanceolate, channelled and open-ended, directed laterally or distolaterally, often raised distally on a smooth, gymnocystal cystid. Mandible lanceolate, 220–245 μm long, slightly longer than the rostrum (Fig. 3B, F).

Ovicell subglobular and slightly prominent, 147–239 (187±34, N = 8) × 262–343 (309±33, N = 8) μm (mean OvL/OvW = 0.60), produced by and continuous with frontal shield of distal zooid, personate, obscuring distal half of the orifice; calcification fabric similar to frontal shield but with smaller pseudopores (diameter 3–8 μm); distal boundary marked by a row of larger pseudopores; proximal margin of gymnocystal calcification forming a raised visor-like band (e.g., Fig. 3B–D). Personate structure of the ovicell with a collar enclosing the ascopore and forming a bridge of two fused flaps between the orifice and the ascopore, producing two secondary openings (Fig. 3D, F, G); secondary orifice transversely elliptical, 71–137 × 180–218 μm; secondary opening over the ascopore trumpet-like (38–52 × 83–145 μm).

Ancestrula tatiform partially overgrown (four spines still visible) and regenerated as an autozooid without avicularium.

###### Etymology.

From the Latin prefix *bi*-, two/double, and the adjective *collaris*, pertaining to the neck, referring to the bridging structure between the orifice and the ascopore appearing as a double collar.

###### Remarks.

Four species with personate ovicells are known to date from the Mediterranean. *Microporellacoronata* (Audouin & Savigny, 1826) differs from the new species in having paired avicularia and a greater number of oral spines, always hidden in ovicellate zooids. *Microporellabrowni* Harmelin, Ostrovsky, Cáceres-Chamizo & Sanner, 2011, *M.genisii* (Audouin & Savigny, 1826), and *M.orientalis* Harmer, 1957 differ in having personate ovicell structures not enclosing the ascopore, and by the denticulation either on the distal or the proximal margin of the orifice.

Among all *Microporella* species known worldwide, the most similar to *M.bicollaris* sp. nov. is the eastern Pacific *M.pontifica* Osburn, 1952 reported from Clarion Island, Galapagos and the Gulf of California. Unfortunately, SEM images are not available for this species, but the original drawing (Osburn 1952: pl. 44, fig. 5) shows the same personate structure of the ovicell observed in *M.bicollaris* sp. nov. However, the new species differs in having a larger avicularium placed more terminally relative to the lateral margin of the zooid, and by the presence of condyles in the orifice. The specimen drawn in Hayward and Ryland (1999: fig. 136D) as *Microporellaciliata* “personate” form of Hincks (1880), also appears similar to *M.bicollaris* sp. nov. However, the illustration in Hincks (1880) appears different, but it is unclear whether Hayward and Ryland (1999) examined any additional material. The north-eastern Atlantic specimens need to be revised to assess their conspecificity with the Mediterranean colonies.

###### Distribution and ecology.

*Microporellabicollaris* sp. nov. is presently known only from Porto Cesareo MPA (Gulf of Taranto, southwestern Apulia, NE Ionian Sea), and the Mazzere submarine cave in the Plemmirio MPA (western Ionian Sea). All colonies are from shallow waters, collected in photophilic algae or found in a semi-dark submarine cave.

##### Microporella ciliata

Taxon classificationAnimaliaCheilostomatidaMicroporellidae

(Pallas, 1766)

1C0DEB92-3BEA-5869-9068-4C04DDC056C7

Fig. 4

Eschara ciliata Pallas, 1766: 38.
Microporella ciliata (Pallas): Kukliński and Taylor 2008: 1895, fig. 1a–i.
Non Microporellaciliata (Pallas): Hayward and McKinney 2002: 83, fig. 37e–j.

###### Examined material.

Italy • 1 dead colony; Ionian Sea, SE Sicily, Gulf of Noto; sample WP120; 36°44'26"N, 15°10'3"E; 50 m; 1996; E. Mollica leg.; dredging; PMC Rosso-Collection I. H. B.85a.

**Figure 4.**
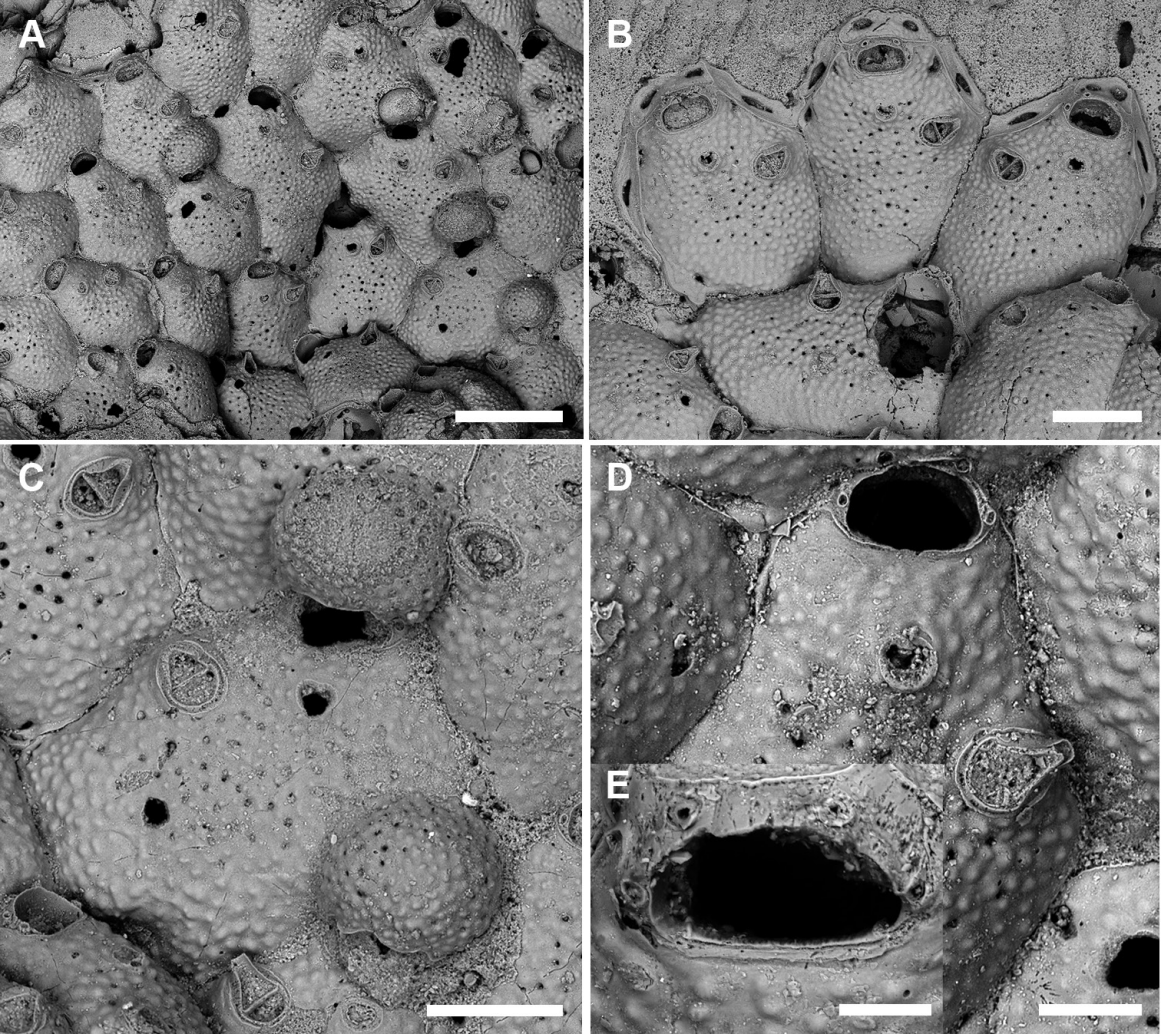
*Microporellaciliata* (Pallas, 1766) from the Ionian Sea, Gulf of Noto, PMC Rosso-Collection I. H. B.85a **A** general view of the colony **B** autozooids at the colony margin showing distal and distolateral pore chamber windows **C** ovicellate autozooids **D** autozooid with the characteristic four, thin, oral spine bases. Note how commonly avicularia regenerate **E** close-up of the orifice showing a series of median denticles and two lateral condyles. Scale bars: 500 µm (**A**); 250 µm (**B**); 200 µm (**C**); 100 µm (**D**); 50 µm **(E)**.

###### Remarks.

The neotype chosen by Kukliński and Taylor (2008) was selected from material collected in 1984 and 2005 at Penta Palummo Bank, a submerged volcanic edifice in the Bay of Naples rising up to about 40 m depth from the surrounding 80 m shelf. Kukliński and Taylor (2008) already suggested that the Mediterranean material overall assigned to *M.ciliata*, might actually correspond to more than one closely related species. This was observed, for instance, in SEM images of north Adriatic specimens in Hayward and McKinney (2002), which differ from the neotype. Following Kukliński and Taylor (2008), Rosso et al. (2010), Chimenz Gusso et al. (2014), and Rosso and Di Martino (2016) remarked on the need for a revision of the material assigned to this species, whose accorded great variability (e.g., Gautier 1962; Hayward and Ryland 1999) is very likely to correspond to a species complex. The effort to disentangle the *ciliata* complex began with the recent description of *Microporellamodesta* Di Martino, Taylor & Gordon, 2020, and is pursued in the present paper with the description of two new species sharing with *M.ciliata* the general appearance and the presence of a single, lateral avicularium as well as non-personate ovicells. Examination of all material available to us, as well as images available in the literature, suggest that *M.ciliata*, as defined by the neotype, is possibly a rare species in the Mediterranean Sea, or at least a species with a very restricted geographical and/or ecological distribution. A similar conclusion was reached by Chimenz Gusso et al. (2014) after revising their material identified as M.cf.ciliata, found associated to *Posidonia* and algae between 5 and 88 m depth in several localities of the Tyrrhenian Sea (i.e., off Latium; Spargiotto Island, NE Sardinia; Ustica and Volcano Islands) and the Sicily Strait (Lampedusa Island). The revision of all specimens originally assigned to *M.ciliata* or M.gr.ciliata in the collections of one of us (AR), originating from several regions and habitats of the Mediterranean Sea, led to the detection of only one colony corresponding with the neotype (Fig. 4). Although the type locality is unknown, it is likely that the original material described by Pallas (1766) was collected in shallow waters from easily accessible substrates, which is not the case for the selected neotype. Colonies of *M.ciliata* recorded from different western Mediterranean localities (Gautier 1962; Zabala 1986, and references therein), Greece (Harmelin 1969) and Turkey (Ünsal 1975), all show great variability and need to be revised to ascertain their identity.

###### Distribution.

Awaiting a general revision of specimens reported from all over the world, focusing on the Mediterranean Sea, the occurrence of *Microporellaciliata* to date is only confirmed in the Gulf of Naples (SE Tyrrhenian Sea) and the Gulf of Noto (W Ionian Sea).

##### Microporella ichnusae sp. nov.

Taxon classificationAnimaliaCheilostomatidaMicroporellidae

B37BD229-D79B-5D09-9676-EE6DBCEB16ED

http://zoobank.org/01CCC2A6-8D65-49F0-8AA9-732ADA5A2ED9

Figs 5 , 6

Microporella sp. A Fraschetti et al. 2010: table 27.

###### Type material.

***Holotype***: Italy • 1 living colony consisting of more than 100 zooids, including some complete and some broken ovicells; Iberian-Provençal Basin, NW Sardinia, Capo Caccia–Isola Piana MPA, Bisbe submarine cave; sample Bisbe 2; 40°34'15"N, 8°12'55"E; 8 m; 2009; V. Di Martino leg.; scuba diving; GSO Biocoenosis; PMC. B30a. 20.11.2020. ***Paratypes***: Italy • 9 living colonies, each consisting of a dozen zooids; Iberian-Provençal Basin, NW Sardinia, Capo Caccia–Isola Piana MPA, Bisbe, Falco and Galatea caves; samples Bisbe 1, Bisbe 2, Falco 2, Galatea 1 and Galatea 2; Bisbe, same details as the holotype; Falco: 40°34'09"N, 8°13'14"E; Galatea: 40°34'09"N, 8°13'54"E; 4–8 m; 2008; V. Di Martino leg.; scuba diving; GSO Biocoenosis; PMC. B30b. 20.11.2020.

###### Diagnosis.

Colony encrusting, multiserial. Autozooid frontal shield densely pustulose and sparsely pseudoporous. Orifice transversely D-shaped; hinge-line smooth with blunt condyles close to corners; four thin oral spines, hidden in ovicellate zooids. Ascopore field semi-elliptical; ascopore opening an arched fissure marked by a distal tongue with radial spines. Avicularium usually single, same level as or proximal to the ascopore, occasionally paired, directed distolaterally; crossbar complete; rostrum lanceolate, channelled. Ovicell non-personate.

###### Description.

**Figure 5.**
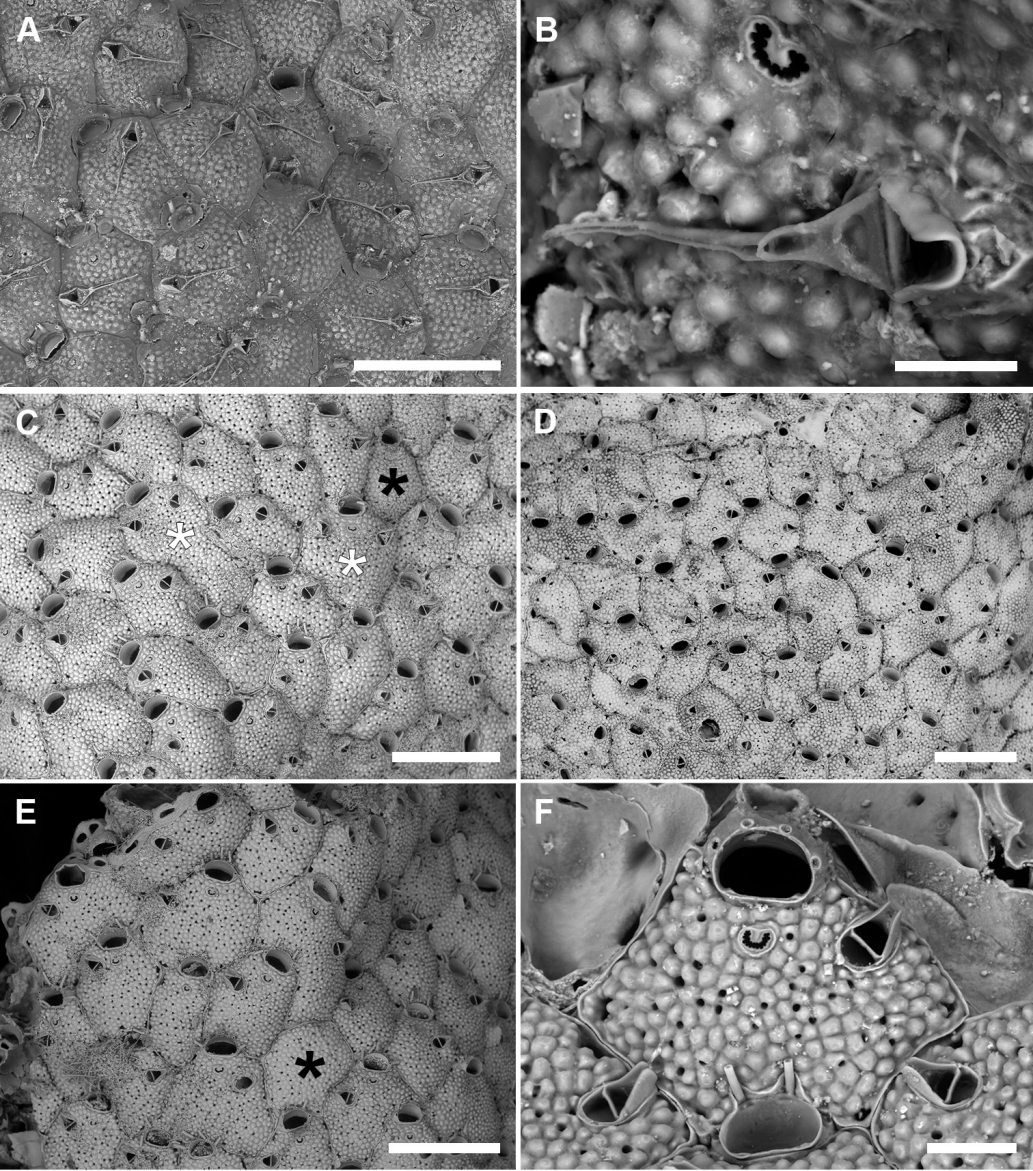
*Microporellaichnusae* sp. nov. from the Iberian-Provençal Basin, NW Sardinia, Capo Caccia-Isola Piana MPA, Falco Cave, Paratype PMC. B30b. 20.11.2020 **A** unbleached colony **B** avicularium with open mandible, showing the channelled rostrum. The straight, short, setiform mandible has a hook at about one-third of its length which clamps it to the rostrum tip **C** bleached autozooids showing size and shape variability. Note that the majority of autozooids has a single avicularium, while few autozooids have paired avicularia (white asterisks) or none (black asterisks) **D** irregularly-shaped autozooids and kenozooids along the contact zone of lobes **E** autozooids and kenozooid (asterisk) near the colony margin with pore-chamber windows visible along exposed lateral walls **F** autozooid at the growing edge showing the morphology of the orifice and four, thin spine bases. Scale bars: 500 µm (**A, C, D, E**); 50 µm (**B**); 100 µm (**F**).

Colony encrusting multiserial, unilaminar (Fig. 5C, D) with zooids arranged in alternate rows often disrupted on particularly irregular substrata; interzooidal communications through 6–8 elliptical pore chamber windows (40–65 × 18–26 µm).

**Figure 6.**
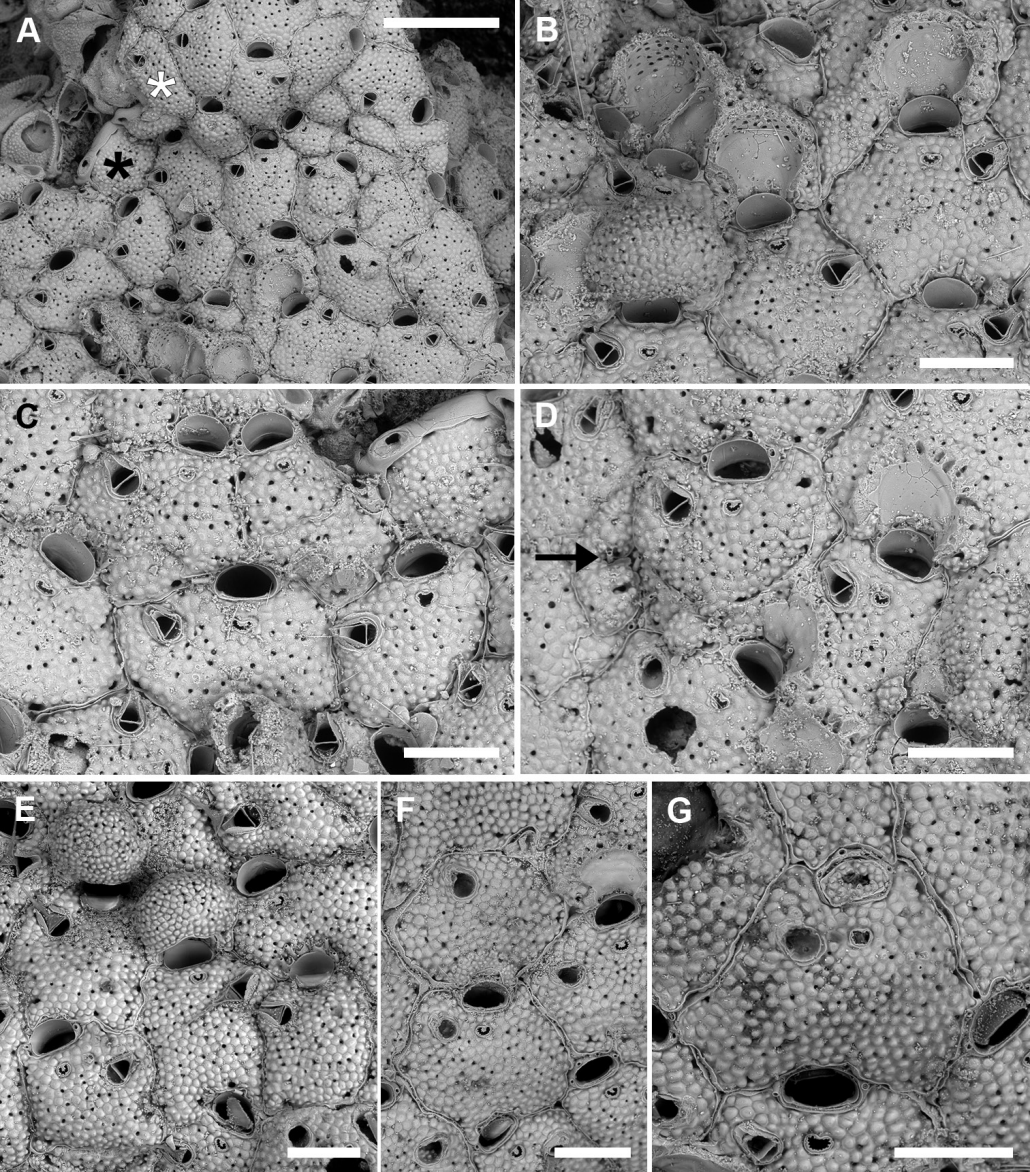
*Microporellaichnusae* sp. nov. from the Iberian-Provençal Basin, NW Sardinia, Capo Caccia-Isola Piana MPA, Bisbe Cave, Holotype PMC. B30a. 20.11.2020 **A** colony portion with broken ovicells, autozooids showing evidences of reparation, and kenozooids with (white asterisk) and without (black asterisk) avicularia **B** autozooids with complete or broken ovicells **C** paired autozooids, seemingly repaired, one lacking avicularium **D** close-up of a colony portion with evidence of zooidal repair. Note the occluded orifice indicated by the partly protruding spines (see arrow) **E** group of zooids (two ovicellate), some with open or closed mandibles and one lacking an avicularium **F** close-up of two zooids. Owing to the absence of an ascopore, which is usually placed at the same level as the avicularium, the upper one is more likely to be a kenozooid equipped with an avicularium than an autozooid with obliterated orifice. Note also the different frontal shield texture of its proximal margin, likely due to ovicell resorption **G** close-up of an autozooid with sealed orifice (central zooid), and an orifice with a thin secondary rim, indicating intramural budding presumably as a result of predation. Scale bars: 500 µm (**A**); 200 µm (**B–G**).

Autozooids usually hexagonal to rhomboidal but sometimes irregularly shaped, 307–587 (434±73, N = 20) × 284–439 (357±59, N = 20 µm) (mean L/W = 1.21), boundaries marked by narrow grooves and raised rims of lateral walls (Figs 5F, 6B, G). Frontal shield slightly convex with polygonal and flat-topped pustules giving a tessellate appearance, and pierced by circular (diameter 6–12 µm), irregularly distributed pseudopores, usually numbering 10–30 but more numerous in some colonies; 2–4 marginal areolae, elliptical to fissure-like, barely visible or distinguishable from pseudopores (Figs 5F, 6B).

Orifice transversely D-shaped, 75–94 (81±5, N = 20) × 109–145 (122±10, N = 20) µm (mean OL/OW = 0.67; mean ZL/OL = 5.33), outlined by a thin, slightly raised rim; hinge-line straight, smooth, with a pair of small triangular, blunt condyles close to corners (Fig. 5F). Oral spines four, occasionally five or six (diameter of the bases 10–14 μm), evenly spaced, the proximal pair located at orifice mid-length; spines hidden in ovicellate autozooids (Fig. 6B, E).

Ascopore field a small and very narrow, transversely semi-elliptical area marked by a thin raised gymnocystal rim, 28–36 × 30–50 μm, located 25–50 μm below the orifice, at the same level as the frontal shield; opening transversely C-shaped, 20–30 × 6–10 μm, with a subcircular tongue projecting from distal edge, and relatively few, tiny, radial denticles.

Avicularium most often single (Figs 5E, F, 6B, D), occasionally paired (Fig. 5C) or absent (Figs 5C, E, 6C), relatively large, 75–120 (98±13, N = 20) × 55–91 (71±11, N = 20) μm (mean AvL/AvW = 1.39), located laterally, on either side, in the distal zooidal half, same level as or proximally to the ascopore (Figs 5B, F, 6B, E) but on irregularly shaped zooids occasionally placed in the proximal part; crossbar complete, thin; rostrum triangular, channelled and open-ended, directed laterally or distolaterally, often distally raised on a smooth, gymnocystal cystid (Fig. 5F). Mandible 160–180 μm long, setiform, with a hook at about one-third of its length that clamps it to the rostrum tip, crossing the whole zooid when open (Fig. 5A, B).

Ovicell subglobular and prominent, 185–241 (214±25, N = 4) × 290–314 (297±11, N = 4) μm (mean OvL/OvW = 0.72), produced by and continuous with frontal shield of distal zooid, obscuring distal part of the orifice; calcification fabric similar to frontal shield but with smaller and more prominent pustules; pseudopores small (diameter 5–10 μm), densely packed at the periphery, absent centrally (Fig. 6B, E).

Kenozooids smaller than or nearly as large as autozooids, lacking openings such as orifices and ascopores but sometimes equipped with avicularium (Fig. 6A, F).

Ancestrula not observed.

###### Etymology.

From *Ichnusa* the Latinized form of the ancient Greek name for Sardinia.

###### Remarks.

Size and shape of autozooids vary remarkably within and between colonies, including dwarf-like autozooids, about half the size of the more regular ones, as well as extremely large and irregularly shaped autozooids, appearing as the result of the fusion of contiguous autozooids (Fig. 5C, E). In this latter case the avicularium can be placed much more proximally than in regular autozooids. Irregularly-shaped autozooids also occur in *M.browni* (Harmelin et al. 2011: fig. 3b). These unusual autozooids and the kenozooids observed in this species seem to be particularly common in zones of contact between colonies or lobes of the same colony, and in damaged areas, also associated with evidences of reparation (Figs 5D, 6A, D), such as regeneration of broken autozooids, patches of calcification to close holes in the frontal shield, or orifices of presumably not functional autozooids (Fig. 6G). Intrazooidal budding, a feature that is common in bryozoans from submarine caves (e.g., Rosso et al. 2020a, 2020b), has been more commonly observed in avicularia (Fig. 6C, D) than autozooids (Fig. 6F, G). The occurrence of ovicells seems rare, observed only on the colony selected as the holotype.

The general appearance of this new species is very similar to *M.ciliata*. However, the orifice in *M.ciliata*, although of comparable size (0.06–0.08 mm long by 0.11–0.15 mm wide), is proportionately shorter, the hinge-line shows a series of median denticles and the two lateral condyles are more prominent and more laterally placed (Fig. 4E; see also Kukliński and Taylor 2008: fig. 1G). The type and position of the oral spines are similar but the number of spines is 4–6 (more commonly four) in *M.ichnusae* sp. nov. and 1–4 (and occasionally lacking in the zone of astogenetic repetition) in *M.ciliata* (Kukliński and Taylor 2008). In *M.ciliata* the frontal avicularium is constantly single, only lacking in the first autozooid budded from the ancestrula, and no kenozooids were reported (Kukliński and Taylor 2008). Furthermore, the ovicells in *M.ciliata* have length comparable with those of *M.ichnusae* sp. nov. but are much narrower.

###### Distribution and ecology.

*Microporellaichnusae* sp. nov. is presently known only from submarine caves in the Capo Caccia-Isola Piana MPA, in NW Sardinia. However, it is possible that some previous records of *M.ciliata*, to date the only *Microporella* species with a single avicularium considered as widespread in the Mediterranean, belong to this species.

##### Microporella modesta

Taxon classificationAnimaliaCheilostomatidaMicroporellidae

Di Martino, Taylor & Gordon, 2020

F3362B34-89CD-5BCD-9F16-D51A30679D19

Fig. 7

Microporella modesta Di Martino et al. 2020a: 23, fig. 11, table 8.
Microporella orientalis Harmer, 1957: Rosso 1996a: table 2.
Microporella ciliata (Pallas): Rosso 1989: tables 3, 4, 6; Di Geronimo et al. 1990: table 1; Di Geronimo et al. 1993b: table 3; Rosso 1996a: table 2; Rosso 1996b: table 1; Hayward and McKinney 2002: 83, fig. 37e–j.
Microporella cf. ciliata (Pallas): Chimenz Gusso et al. 2014: 189, fig. 101a–f.
Microporella gr. ciliata (Pallas): Rosso et al. 2019a: table 1.

###### Examined material.

Italy • 30 dead colony fragments, 8 of which bilaminar, 1 pseudovinculariform and the majority encrusting on *Cellaria* internodes; Ionian Sea, E Sicily, Ciclopi Islands MPA; Ciclopi 2000 cruise; sample 2G, 8I, 9G, 12E, 12F, 12G; 37°34'4"N, 15°10'51"E; 63–95 m; Jul. 2000; DC, DE–DL, DL Biocoenoses; A. Rosso leg.; dredging; PMC Rosso-Collection I. H. B.86a. Italy • 1 living colony, Ionian Sea, E Sicily, Ciclopi Islands MPA; sample SM1Z25; 37°38'17"N, 15°10'53"E; 25 m; Jun. 2015; R. Leonardi leg.; scuba diving; IA Biocoenosis; PMC Rosso-Collection I. H. B.86a1. Italy • 2 living colonies fouling on a plastic bag; Ionian Sea, E Sicily, Gulf of Ognina, North of Catania; 37°31'52"N, 15°6'59"E; 4 m; 11 Feb. 2012; V. Grado leg.; scuba diving; PMC Rosso-Collection I. H. B.86b. Italy • 15 living colonies on *Posidonia* leaves; Ionian Sea, E Sicily, S of the Gulf of Catania, Castelluccio; 37°18'32"N, 15°7'59"E; beached; 6 Feb. 2019; A. Rosso leg.; hand-collected; PMC Rosso-Collection I. H. B.86c. Italy • 3 living and 1 dead colony fragments; Ionian Sea, SE Sicily, Plemmirio MPA, Granchi submarine cave; sample GR1; 37°00'18"N, 15°18'35"E; 23 m; 14 Sep. 2009; V. Di Martino leg.; scuba diving; C and GSO Biocoenoses; PMC Rosso-Collection I. H. B.86d. Italy • 16 living and 32 dead colony fragments, nearly all on *Cellaria* internodes, Ionian Sea, SE Sicily, Gulf of Noto; 36°41'45"–36°57'47"N, 15°8'35"–15°20'00"E; PS/81 cruise; samples CR1, 2XB, 4C, 4X, 9B, 9C, 9D, 10C, 11E; 45 m (living), 44–120 m (dead); I. Di Geronimo leg.; dredging; and 25 living and 25 dead colony fragments, nearly all on *Cellaria* internodes; Noto 1996 cruise; samples 3C, 5E, 7E, 10G, 10I, WP120; 20–82 m (living), 90–107 m (dead); 1996; E. Mollica leg.; dredging; C, DC, DE and DL Biocoenoses; PMC Rosso-Collection I. H. B.86e. Italy • 2 living colonies, Ionian Sea, Gulf of Taranto, Amendolara Bank; samples 1D and 5D; 39°51'42"–39°52'54"N, 16°42'00"–16°43'24"E; 30–40 m; Jun. 1991; R. Sanfilippo leg.; dredging; DC Biocoenosis; PMC Rosso-Collection I. H. B.86f. Italy • 4 living and 3 dead colonies; Sicily Strait, Pelagie Islands MPA, Lampedusa Island; submarine caves: Taccio Vecchio I, 35°31'29"N, 12°35'58"E, 20 m; Grotta della Madonna, 35°30'2"N, 12°33'25"E, 15 m; Grotta dello Scoglio di Fora, 35°30'25"N, 12°33'33"E, 10 m; Jun. 2009; V. Di Martino leg.; scuba diving; C and GSO Biocoenoses; PMC Rosso-Collection I. H. B.86g. Italy • about 100 living colonies, mostly on *Posidonia* leaves, soft algae, light calcified *Peissonnelia* spp. and calcified thin-branched geniculate corallines; Sicily Strait, Egadi Islands, Marettimo Island; sample ECE5; 37°56'59"N, 12°3'56"E; 8 m; summer 2007; A. Sinagra leg.; IA and HP Biocoenoses; scuba diving; PMC Rosso-Collection I. H. B.86h. Italy • 6 living and 2 dead colony fragments, 2 of which bilaminar; southern Tyrrhenian Sea, SW Ustica, Apollo Bank; 38°42'19"N, 13°7'58"E; 60 m; Jun. 1986, dredging and scuba diving; *Laminariarodriguezii* Bornet, 1888 seagrass and associated DC; PMC Rosso-Collection I. H. B.86i. Italy • 7 living colonies; Iberian-Provençal Basin, Asinara MPA; samples PSE and PSI1; 41°6'59"N, 8°18'6"E; 5–15 m; A. Sinagra leg.; scuba diving; IA Biocoenosis; PMC Rosso-Collection I. H. B.86j. Italy • 1 dead colony, southern Adriatic Sea, off Apulia, Bari canyon; sample 1B1; 41°17'29"N, 17°9'14"E; 280 m; 29 May 2012; F. Mastrototaro leg.; dredging; PMC Rosso-Collection I. H. B.86k.

**Figure 7.**
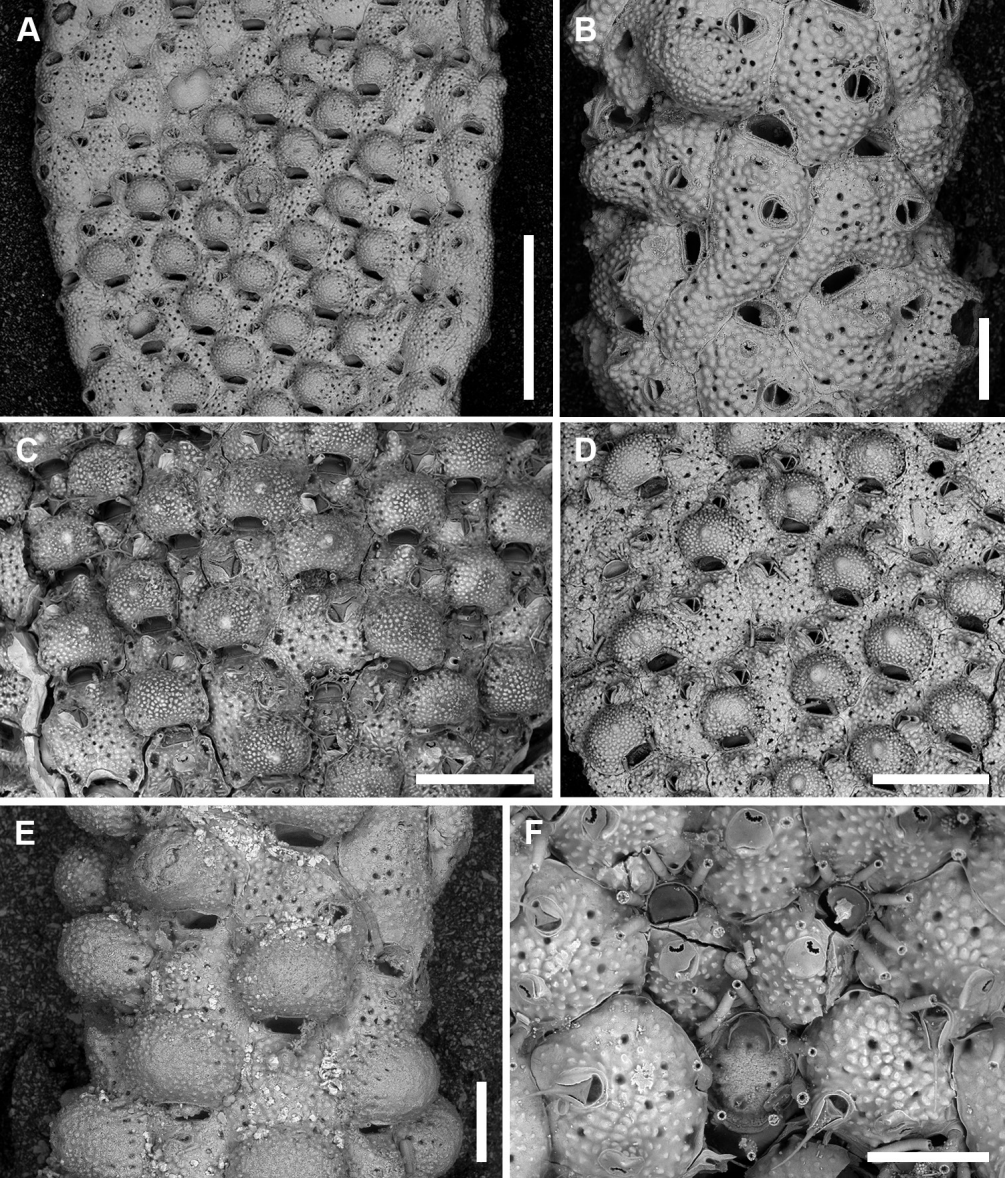
*Microporellamodesta* Di Martino, Taylor & Gordon, 2020 from several localities **A** Ionian Sea, Ciclopi 2000, PMC Rosso-Collection I. H. B.86a **B** Ionian Sea, Gulf of Noto, PMC Rosso-Collection I. H. B.86e **C** Sicily Strait, Egadi Islands, PMC Rosso-Collection I. H. B.86h **D** Ionian Sea, Castelluccio, PMC Rosso-Collection I. H. B.86c **F** Iberian-Provençal Basin, Asinara MPA, PMC Rosso-Collection I. H. B.86j **A** bilaminar branch with elongate autozooids along branch edge and a cluster of ovicells **B** apparently cylindrical branch resulting from a colony encrusting an internode of *Cellaria*. Note the formation of irregularly elongate autozooids **C** unbleached colony encrusting a soft algal frond. Prominent avicularian rostra and mucros associated with ascopore contribute to the spiky appearance of the colony. Note the co-occurrence of ovicells with and without frontal tubercles **D** bleached colony on algae with ovicells with different degrees of frontal tubercle development **E** ovicellate zooids of a tubular “pseudovinculariiform” colony originally developed on a thin ephemeral substratum **F** originally tatiform ancestrula regenerated as a kenozooid, budding two distolateral autozooids lacking avicularia. Scale bars: 1 mm (**A**); 200 µm (**B, E, F**); 500 µm (**C, D**).

###### Remarks.

*Microporellamodesta* has been recently established (Di Martino et al. 2020a) for some Mediterranean specimens collected off Algeria and growing as erect, narrow, bilaminar fronds. However, SEM images in Hayward and McKinney (2002) and Chimenz Gusso et al. (2014) reveal that a large part of the figured Mediterranean specimens seem to be conspecific with *M.modesta*, although none of them has been described as erect bilaminar. This is also the case for most colonies and colony fragments in the collection of one us (AR), mostly previously assigned to *M.ciliata*, which allowed us to assess the intraspecific variability of *M.modesta*.

Strap-like branches including up to 11 or 12 longitudinal series of zooids occur only occasionally (Fig. 7A), while bilaminar arched fronds narrowing proximally are rare, and encrusting sheets are the most common. Branches are always broken off so that the way they form from the putative encrusting base remains unknown. Lateral edges often consist of extremely elongate autozooids covering the length of three regular autozooids, with the proximal two thirds about half the width of the distal part (Fig. 7A). Apparently cylindrical branches result from colonies encrusting thin, cylindrical substrata (Fig. 7B, E), either mineralised and persistent (mostly internodes of *Cellaria* in deep-water samples, and geniculate coralline algae in shallow-water samples), or organic and ephemeral. In this latter case, the resulting pseudovinculariiform colonies are found in the sediment as hollow tubular fragments after substratum decay. A great number of colonies were observed encrusting soft algae, small *Peissonnelia* spp., and leaves of *Posidoniaoceanica* (Linnaeus) Delile 1813, usually developing relatively small colonies, occasionally reaching 1–2 cm^2^.

In addition to colony morphology, variability includes also autozooidal characters. The orificial condyles were less prominent and more laterally placed in specimens from the Apollo Bank; the number of spines is usually five in encrusting colonies from the Ionian Sea (e.g., Amendolara and Ognina), but up to six or seven on some autozooids in colonies from the Sicily Strait (e.g., Madonna cave); spines also tend to be thicker, and the proximal pair more visible on ovicellate autozooids (e.g., in colonies from caves of the Ionian Sea and Sicily Strait) than in the holotype; a prominent central umbo can develop on the ovicell of some zooids (Fig. 7C, D). As in *Microporellaichnusae* sp. nov., irregularly shaped elongated autozooids develop when lobes of a colony join, mostly in colonies on cylindrical substrata (Fig. 7B). Intrazooidal budding frequently occurs in avicularia, while repair of autozooids was rarely observed.

The ancestrula was observed for the first time only in two colonies (sample PSI1 and ECE5), seemingly because it is soon overgrown. It is tatiform, with 10–12 spines surrounding a very thin, raised rim without apparent cryptocyst. It is rebudded as a kenozooid with a row of at least eight pores in one case (Fig. 7F). A comparable structure was figured in Kukliński and Taylor (2008) for *M.ciliata* and can be also observed in *M.browni* from the Maldive Islands (https://www.univie.ac.at/Palaeontologie/Sammlung/Bryozoa/Maldive_Islands/Cheilostomata/Microporellidae/Microporella/Microporella-browni.html). The ancestrula buds two distolateral autozooids with 5–7 oral spines.

Ovicells are very numerous in this species, occurring in the majority of autozooids in some colonies (Fig. 7A, C, E). Ovicell tubercles when developed, along with the mucro associated to the ascopore, and the raised rostrum of the avicularium give to the colony a characteristic knobby or spiky appearance.

The majority of specimens recorded from the Mediterranean previously assigned to *M.ciliata*, as well as some isolated colony fragments attributed to *M.orientalis* seem to belong to *M.modesta*.

###### Distribution and ecology.

*Microporellamodesta* was previously reported exclusively from off Algeria (Di Martino et al. 2020a), but the material studied here as well as specimens figured in recent literature (Hayward and McKinney 2002; Chimenz Gusso et al. 2014) widen its geographical distribution. The species appears to be widely distributed in the Mediterranean where it occurs in the Iberian-Provençal basin, the Tyrrhenian, Ionian, and Adriatic seas as well as in the Sicily Strait. The species seems widely distributed in shallow shelf habitats with preference for the vegetate bottoms of the infralittoral zone and the Coralligenous Biocoenosis, with the possibility to extend into semi-dark caves. Findings from deeper (mid-shelf) particulate bottoms are largely represented by dead colonies/fragments possibly indicating a displacement.

##### Microporella pachyspina sp. nov.

Taxon classificationAnimaliaCheilostomatidaMicroporellidae

519057F2-4BF5-54DE-97FB-33BC254677C7

http://zoobank.org/01E293A7-7645-4D29-B3D3-70905E995CE8

Fig. 8

###### Type material.

***Holotype***: Italy • 1 living colony consisting of about 50 zooids, several fertile; Sicily Strait, Egadi Islands, Marettimo Island; 37°56'59"N, 12°3'56"E; sample ECE5; 8 m; summer 2007; A. Sinagra leg.; IA and HP Biocoenoses; scuba diving; PMC. B31a.3.12.2020. ***Paratypes***: Italy • ECE5, 2 living, fertile colonies, one including the ancestrula on a *Posidoniaoceanica* leaf; same details as the holotype; PMC. B31b. 3.12.2020.

###### Other material.

Italy • a few living colonies, Sicily Strait, Egadi Islands, Marettimo Island; 37°56'43"N, 12°5'3"E; sample EBE4; 19 m; summer 2007; A. Sinagra leg.; IA-HP Biocoenoses; scuba diving; PMC Rosso Collection I. H. B.87a.

###### Diagnosis.

Colony encrusting, multiserial. Autozooid frontal shield granular and centrally pseudoporous. Orifice transversely D-shaped; hinge-line smooth with rectangular condyles at corners; five (more commonly) to eight oral spines, the proximalmost pair placed slightly below the orifice hinge-line and very large in diameter. Ascopore field reniform to elliptical, developing a mucro proximally; ascopore opening transversely C-shaped, with tongue and radial spines. Avicularium single, located at half zooidal length, directed distolaterally; crossbar complete; rostrum triangular, channelled. Ovicell produced by distal autozooid, non-personate.

###### Description.

Colony encrusting, multiserial, unilaminar, forming subcircular patches less than 1 cm in diameter, consisting of several tens of zooids, typically on *Posidonia* leaves; interzooidal communications through pore chamber windows along lateral walls (44–99 × 12–20 μm), two elliptical pairs placed proximolaterally and distolaterally, and a single distal one more rounded.

**Figure 8.**
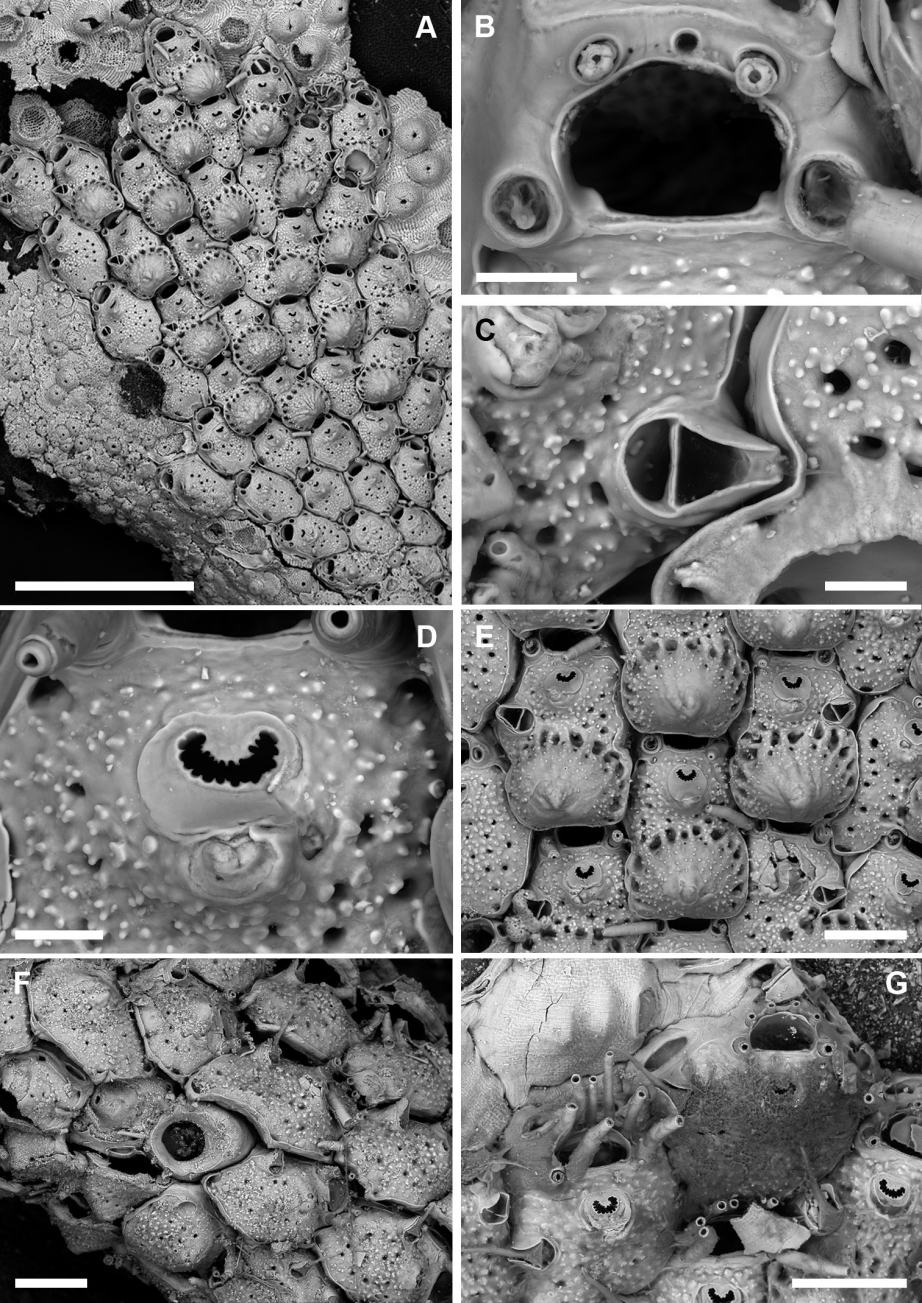
*Microporellapachyspina* sp. nov. from Sicily Strait, Egadi Islands **A–E** holotype PMC. B31a.3.12.2020 **F, G** paratype PMC. B31b. 3.12.2020 **A** colony general view **B** close-up of orifice with proximolateral rectangular condyles, smooth hinge-line, and five oral spine bases. Note the giant proximolateral pair of spine bases located at hinge-line level and at some distance from the thinner, distal ones **C** close-up of avicularium with truncated, channelled rostrum projecting laterally outside its edge affecting the shape of the adjacent zooid margin **D** close-up of ascopore with proximal mucro **E** ovicells smooth and imperforate centrally, finely granular and with a row of large pores peripherally. Radial buttresses between marginal pores converge towards the median umbo **F** Ancestrula budding one distal and two distolateral autozooids. Two larger, proximolateral, curved autozooids complete the periancestrular region **G** autozooids from the colony growing margin with six or seven oral spines. Scale bars: 1 mm (**A**); 50 µm (**B–D**); 200 µm (**E–G**).

Autozooids hexagonal, 374–510 (442±50, N = 24) × 257–346 (290±31, N = 24) µm (mean L/W = 1.52), distinct with interzooidal boundaries marked by deep grooves between salient vertical walls (Fig. 8A), often exposing the smooth, lateral gymnocyst. Frontal shield nearly flat proximally, slightly convex disto-centrally, finely to coarsely granular and pseudoporous (Fig. 8C, D); granules 5–10 µm in diameter, irregularly spaced; 6–18 pseudopores, circular (7–25 μm in diameter), irregularly arranged, mostly centrally in the proximal half of the zooid; 3–6 marginal areolae, usually visible at zooidal corners, those placed proximally and laterally fissure-like (up to 90 μm long), the single or paired distal ones rounded (20 µm in diameter).

Orifice transversely D-shaped, 75–93 (84±6, N = 15) × 89–127 µm (108±12, N = 15) (mean OL/OW = 0.78; mean ZL/OL = 5.17), outlined by a thin and smooth raised rim; hinge-line straight, smooth, with a pair of rectangular condyles at corners (Fig. 8B). Five (Fig. 8B) (occasionally 6–8: Fig. 8G) oral spines; the proximalmost pair robust, the base 36–51 µm in diameter, horn-shaped, terminally tapering and bending, placed slightly below the level of the orifice hinge-line, persisting in ovicellate autozooids (Fig. 8E); the three (occasionally 4–6) distal spines thinner (base diameter 13–32 μm).

Ascopore field a reniform to elliptical area of smooth gymnocystal calcification (39–78 × 45–102 μm), more extensive proximally, developing a pointed, upward directed mucro not concealing the ascopore opening, placed 30–60 μm below the orifice, slightly depressed relative to the adjacent frontal shield (Fig. 8D); opening transversely C-shaped, 32–64 × 7–18 μm, with subcircular tongue projecting from distal edge and radial, tiny denticles.

Avicularium single, often absent (e.g., 40% of zooids without avicularium in a colony of 42 zooids), moderately large, 76–115 (94±10, N = 33) × 48–78 μm (63±9, N = 33) (mean AvL/AvW = 1.50), located laterally, on either side, at about half zooidal length; crossbar complete; rostrum short, rounded triangular, channelled, directed distolaterally, often raised distally on a smooth, gymnocystal cystid (Fig. 8C). Mandible 143–224 μm long, pointed, with a hook at about one-third of its length that clamps it to the rostrum tip, lying proximally to ascopore when open (Fig. 8G).

Ovicell non-personate, subglobular, prominent, 216–320 (251±23, N = 20) × 241–312 (288±22, N = 20) μm (mean OvL/OvW = 0.87), obscuring half to two-thirds of the zooidal orifice, formed by and continuous with frontal shield of distal zooid (Fig. 8E) (occasionally of two zooids), lateral gymnocystal margins raised, exceeding boundaries of the autozooid on which it lies; calcification fabric similar to frontal shield but with smaller and more widely-spaced granules, sometimes completely smooth centrally and with a rounded mucro (Fig. 8A, E); imperforate except for 12–18 large pseudopores aligned in a peripheral row, closely and evenly spaced, separated by radial ridges, rounded quadrangular, 17–42 μm in diameter, plus an additional, discontinuous inner row of 4–6 smaller, circular pseudopores (5–10 μm).

Ancestrula tatiform, oval (300 × 218 μm), gymnocyst moderately developed, more extensive proximally (Fig. 8F); opesia subcircular, surrounded by a well-developed, smooth cryptocyst, more extensive proximally, narrowing distally, outlined by a thin elevated rim indented by ten gymnocystal spines (six distal, two median, two proximal). Ancestrula budding two distolateral autozooids, followed by two lateral and two proximolateral ones.

###### Etymology.

From the Greek *pachys*, meaning thick, and the Latin *spina* meaning spine, referring to the robust proximalmost pair of oral spines.

###### Remarks.

The main diagnostic character of *Microporellapachyspina* sp. nov. is the great size of the proximalmost pair of oral spines, as well as their position, halfway below the level of the orifice hinge-line. Among *Microporella* species known worldwide, *M.alaskana* Dick & Ross, 1988 from the eastern Pacific, *M.echinata* Androsova, 1958, and *M.trigonellata* Suwa & Mawatari, 1998, both from off Japan, share similar features. In *M.alaskana* the proximalmost pair of spines are larger in diameter compared to the remaining spines but they are placed more distally compared to the new species, approximately at orifice mid-length (Dick and Ross 1988); in addition, this species has paired avicularia, the ascopore is placed very close to the orifice hinge-line, and there is a prominent umbo centrally on the frontal shield (Dick and Ross 1988). *Microporellaechinata* differs in having an evenly pseudoporous frontal shield, with pseudopores visibly larger, and tubercular (Mawatari et al. 1991). *Microporellatrigonellata* shows the same number, relative size, arrangement and position of spines but differs from the new species in having the avicularium placed distolaterally, directed distally, with a pointed, non-channelled rostrum; also, the ridges and grooves on the ovicells are distinctly defined and more marked, and the ancestrula has a narrower proximal gymnocyst (Suwa and Mawatari 1998).

The general appearance of those zooids lacking avicularia in *M.pachyspina* sp. nov. reminds those of *Fenestrulinajoannae* (Calvet, 1902), which are also similar in having the proximalmost pair of spines long, robust and rounded, non-stellate pseudopores sparse on the frontal shield, centrally smooth ovicells, sometimes with peripheral radial ridges, developing a mucro (Chimenz Gusso et al. 2014: 165, fig. 82a–c). This latter species, originally described as *Microporella* by Calvet (1902), was reassigned to *Fenestrulina* by Gautier (1962: 171) apparently based on a suggestion made by Hastings without any supporting statement, instead highlighting the different type of pseudopores (non-stellate) compared with those of the type species of the genus *Fenestrulinamalusii* Audouin, 1826. Subsequent authors followed Gautier (1962). *Fenestrulina* species have large, stellate pseudopores mostly occupying the area of the frontal shield between the ascopore and the orifice, a sector that is usually imperforate in *Microporella*. Based on these observations, *Fenestrulinajoannae* seems to have more affinities with *Microporella* and here we suggest its displacement.

Dry specimens on organic substrates (i.e., *Posidonia* leaves) appear with the zooids disconnected or almost disconnected, giving to the colony a slightly disjunct appearance because the zooids were less packed hence exposing a more extensive, smooth gymnocyst laterally (Fig. 8F). This loose packing is a common adaptation in species growing on flexible substrates to reduce the potential breakage of the zooidal skeletons.

###### Distribution and ecology.

Presently known only from shallow waters off Egadi Islands, at the western limit of the Sicily Strait in the Mediterranean Sea, associated with *Posidonia* meadows and the Infralittoral Algae Biocoenosis.

##### Microporella

Taxon classificationAnimaliaCheilostomatidaMicroporellidae

sp. A

5360DFB1-1A10-5591-98EB-6F5D4542B501

Fig. 9

###### Examined material.

Italy • 1 dead colony fragment consisting of ca. 14 zooids (some incomplete), none fertile; Tyrrhenian Sea, Palinuro Cape, Scaletta submarine cave; sediment sample; 40°1'35"N, 15°16'7"E; 46 m; 14 Sep. 2009; R. Leonardi leg.; scuba diving; PMC Rosso Collection I. H. B.88a.

###### Description.

Colony encrusting, multiserial, unilaminar.

**Figure 9.**
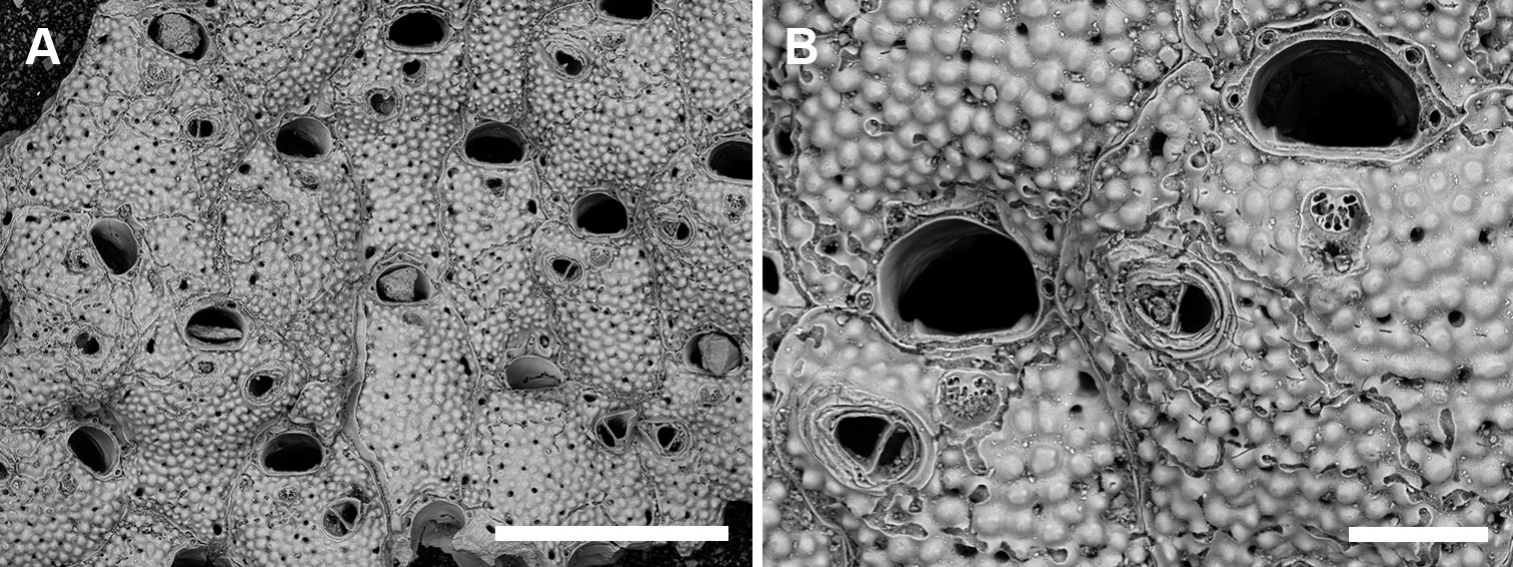
*Microporella* sp. A from Palinuro Cape, Scaletta cave PMC Rosso Collection I. H. B.88a **A** irregularly shaped autozooids with inconstant avicularia **B** close-up of two zooids with details of the orifice, the lateral condyles on the hinge-line, four or five thin oral spines and the ascopore divided by thin radial septa. Scale bars: 500 µm (**A**); 100 µm (**B**).

Autozooids irregularly polygonal, rounded, 435–676 (510±80, N = 7) × 255–427 µm (342±68, N = 7) (mean L/W = 1.49), distinct, with interzooidal boundaries marked by a narrow, raised, gymnocystal rim (Fig. 9A). Frontal shield nearly flat to slightly convex, densely and coarsely granular and irregularly pseudoporous; granules 5–25 µm in diameter; 20–30 pseudopores, circular (5–12 μm in diameter), sparse in the proximal two-thirds of the zooid; 4–6 marginal areolae, usually visible at zooidal corners, circular to elliptical (10–40 µm long).

Orifice transversely D-shaped, 90–107 (94±5, N = 10) × 118–143 (132±9, N = 10) µm (mean OL/OW = 0.71; mean ZL/OL = 5.43); hinge-line straight, smooth to slightly crenulated; in each corner a short, blunt, triangular condyle directed distally (Fig. 9B). Oral spine bases four or five, 10–18 µm in diameter, evenly spaced, the proximalmost pair at about one-third of orifice length (Fig. 9B).

Ascopore field a narrow, elliptical area of smooth gymnocystal calcification (33–44 × 39–55 μm), placed 22–30 μm below the orifice, slightly depressed relative to the adjacent frontal shield; ascopore opening divided by thin radial septa, usually with a distinct tongue extending proximally from the distal edge (Fig. 9B).

Avicularium single, sometimes absent (two out of 14 zooids without avicularium in the fragment available), 93–123 (107±12, N = 9) × 70–87 (79±6, N = 9) μm (mean AvL/AvW = 1.36), located distolaterally, on either side; crossbar complete; rostrum short, triangular, not channelled, directed distolaterally, sometimes slightly raised distally (Fig. 9A, B). Mandible, ovicells and ancestrula not observed. Subsequent intramural budding observed in avicularia.

###### Remarks.

This species differs from its Mediterranean congeners in having a finely reticulate ascopore but it is left in open nomenclature owing to the availability of a single, infertile colony fragment. Similar ascopores can be found in *M.arctica* Norman, 1903 from Norway, *M.ketchikanensis* Dick, Grischenko & Mawatari, 2005 from Alaska, *M.santabarbarensis* Soule, Chaney & Morris, 2004 from southern California, and *M.stellata* (Verril, 1879) from Maine, USA. *Microporellaarctica* differs from *Microporella* sp. A in having a finely granular frontal shield pierced by a greater number of marginal areolae that are always very distinct from pseudopores, in the lack of oral spines, and in having a smooth gymnocystal area laterally and proximally to the orifice that is continuous with the gymnocyst of the ascopore field (Kukliński and Taylor 2008). The ascopores of both *M.ketchikanensis* and *M.santabarbarensis* have a similar, delicate cribrate aspect but lack the distal tongue extending from the distal edge (Dick et al. 2005; Soule et al. 2004). *Microporellastellata* differs in having only two oral spines and a proximal orifice margin with broad, rectangular condyles (Winston et al. 2000).

###### Distribution and ecology.

Presently known only from the Palinuro Peninsula, along the Tyrrhenian coast of Campania (southern Italy). A dead colony was collected from the biogenic muddy sediment covering the floor of a completely dark sector of the Scaletta submarine cave, at 46 m depth where the colony presumably lived.

##### Microporella verrucosa

Taxon classificationAnimaliaCheilostomatidaMicroporellidae

(Peach, 1868)

51F39AB3-0418-5A95-A2A0-AE1A1529BDFC

Figs 10 , 11

Eschara verrucosa Peach, 1868: 116.
Diporula verrucosa (Peach): Hincks, 1880: 220, pl. 31, figs 1, 2; Gautier, 1962: 176; Zabala, 1986: 501, fig. 174, pl. 15A, B; Hayward and Ryland, 1979: 226, fig. 97; Hayward and Ryland, 1999: 302, figs 138C, D, 139; André et al. 2014: 225, 5 figs; Rosso et al. 2014: table 2, fig. 3A–C.
Microporella (Diporula) verrucosa (Peach): Neviani, 1896a: 105; 1896b: 24.

###### Examined material.

Italy • 2 colonies and 10 fragments (living), 17 colonies and 62 fragments (dead), some very large, some regenerated and twisted; Ionian Sea, SE Sicily, Ciclopi Islands MPA; Ciclopi 2000 cruise; samples 2G, 3H, 4E, 6H, 8F, 8H, 8I, 9G, 10G, 12E, 12F, 12G, 13H, 14G; 37°32'39"–37°34'31"N, 15°8'58"–15°11'1"E; 63–92.5 m; Jul. 2000; A. Rosso leg.; dredging; C, DC, DE, and DL Biocoenoses; PMC. Rosso Collection I.H. B-17a. Italy • 11 living and 33 dead colonies/large fragments, 1 dead colony including the base; off Acitrezza; sample AC/L, AC/1B; coordinates unknown; 50 and 110 m; 1980; I. Di Geronimo leg.; dredging; pre-Coralligenous and DL Biocoenoses; PMC. Rosso Collection I.H. B-17a1. Italy • 12 living and 315 dead colonies/fragments; Ionian Sea, Gulf of Noto; 36°41'45"–36°57'47"N, 15°8'35"–15°20'00"E; PS/81 cruise; samples 2C, 4X, 9D (living) and samples 2B, 2C, 2XA, 2XB, 4C, 4C1, 4X, 6D, 9C, 9D, 10C, 11E (dead); Jul. 1981; I. Di Geronimo leg.; dredging; DC and DL Biocoenoses; and 11 living colonies; Noto 1996 cruise; samples 8I, 10G, 10H; 77–82 m; 1996; E. Mollica leg.; dredging; DE and DL Biocoenoses; PMC. Rosso Collection I.H. B-17b. Italy • 18 dead colonies; Ionian Sea, Gulf of Catania; sample LCT69; 37°18'42"N, 15°14'24"E; 90 m; Jul. 1980; I. Di Geronimo leg.; dredging; DL Biocoenosis; PMC. Rosso Collection I.H. B-17c. Italy • 4 dead colonies; Ionian Sea, Gulf of Taranto, Amendolara Bank; samples 1D and 5D; 39°51'42"–39°52'54"N, 16°42'00"–16°43'24"E; 30–40 m; Jun. 1991; R. Sanfilippo leg.; dredging; DC Biocoenosis; PMC. Rosso Collection I.H. B-17g. Italy • 77 dead colonies and fragments; southern Tyrrhenian Sea, SW Ustica, Apollo Bank; 38°42'19"N, 13°7'58"E; 60 m; Jun. 1986, dredging; *Laminariarodriguezii* Bornet, 1888 seagrass and associated DC Biocoenosis; PMC. Rosso Collection I.H. B-17d. Italy • 2 living colonies; Messina Strait; coordinates unknown; 65 m; 1990; S. Giacobbe leg.; dredging; no Biocoenosis information; PMC. Rosso Collection I.H. B-17g. France • 50 dead colonies; Iberian-Provençal Basin, Corsica, off Calvì; sample CL74; 42°47'31"N, 9°8'10"E; 150–110 m; G. Fredj leg.; dredging; DL Biocoenosis; PMC. Rosso Collection F.H. B-17e. Greece • 4 dead colonies, Aegean Sea, Lesvos Island, Agios Vasilios cave; samples AV1 and AV2; 38°58'8"N, 26°32'28"E; 30 m; V. Gerovasileiou leg.; scuba diving; GSO and GO Biocoenoses; PMC. Rosso Collection GR.H. B-17f.

###### Description.

**Figure 10.**
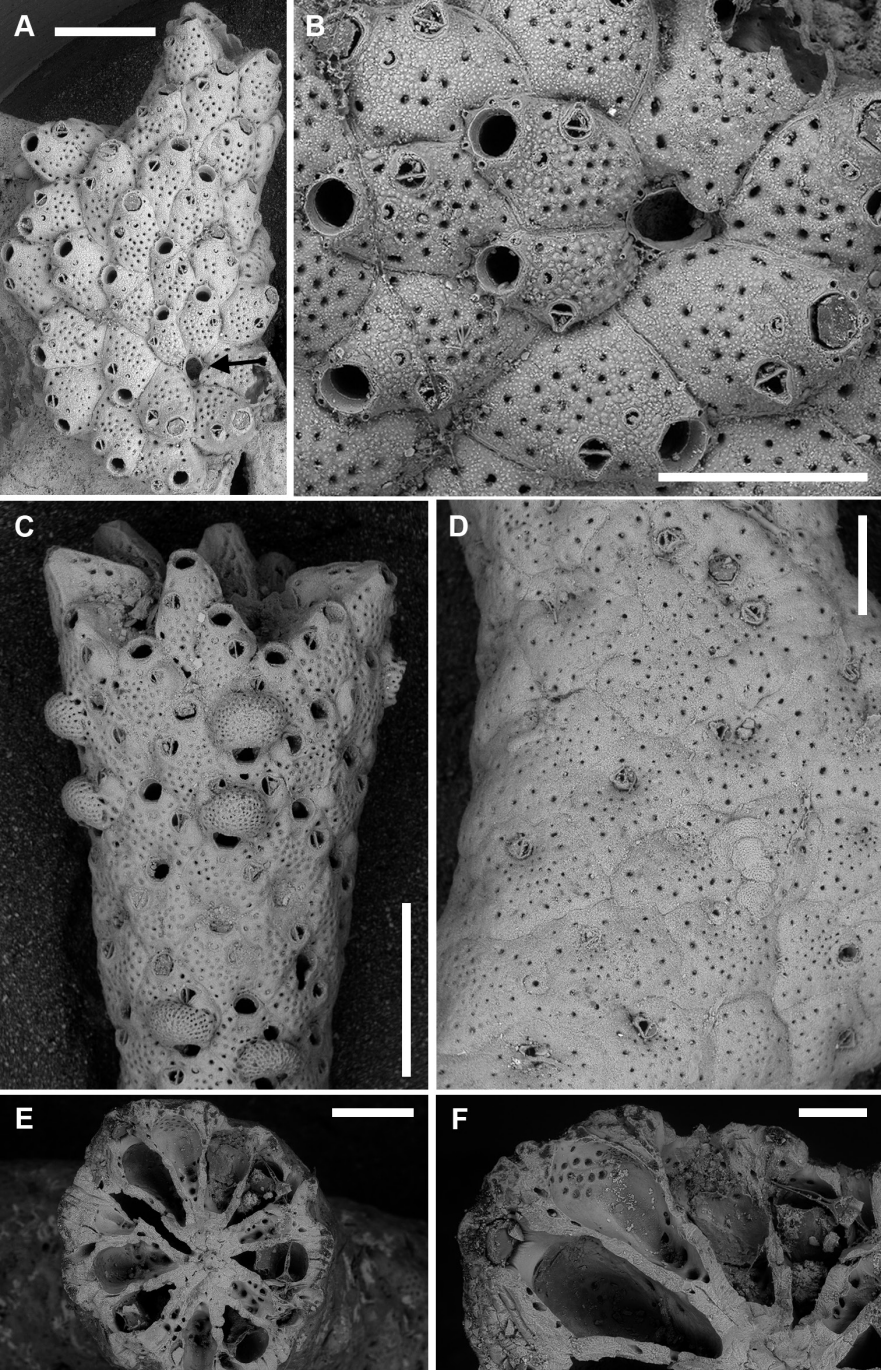
*Microporellaverrucosa* (Peach, 1868) from the Ionian Sea, Ciclopi Islands MPA**A, B**PMC. Rosso Collection I.H. B-17a **C–F**PMC. Rosso Collection I.H. B-17a1 **A** young colony showing the encrusting portion with ancestrula (arrow) and starting developing branch (top right) **B** partly overgrown ancestrula and periancestrular autozooids **C** growing tip of a distally enlarging branch **D** old (proximal) branch portion showing secondary calcification obliterating orifices, sometimes only frontal avicularia are still visible **E** transverse section of a branch showing the thick walls converging towards the centre of the branch **F** close-up of wedge-shaped polypide cavities visible in transverse section. Scale bars: 500 µm (**A, B, D, E**); 1 mm (**C**); 200 µm (**F**).

Colony erect, rigid, branched, with a limited number of relatively spaced-out bifurcations, a few cm long, raising from an encrusting basal portion (Fig. 10A), extending up to 3 mm around the main stem.

Branches cylindrical, often flattened at bifurcations (Fig. 10C, E), consisting of 9–16 longitudinal rows of zooids, alternating very regularly to simulate an helicoidal pattern; branch diameter 10–15 mm (exceptionally up to 20 mm), becoming thicker and stouter in older portions of the colony, near the encrusting base, owing to secondary calcification.

Encrusting base unilaminar, multiserial (Fig. 10A), formed by autozooids similar to those of the erect branches (Fig. 10B) but with a greater number of oral spines (5–7, commonly six) and, subordinately, autozooids with occluded orifices (not functional) and/or kenozooids, often bearing an adventitious avicularium as those associated with autozooids. Interzooidal communications via basal pore-chambers in the encrusting portion (see Rosso et al. 2014: fig. 3A) and via multiporous septula in the erect branches (Fig. 10C).

**Figure 11.**
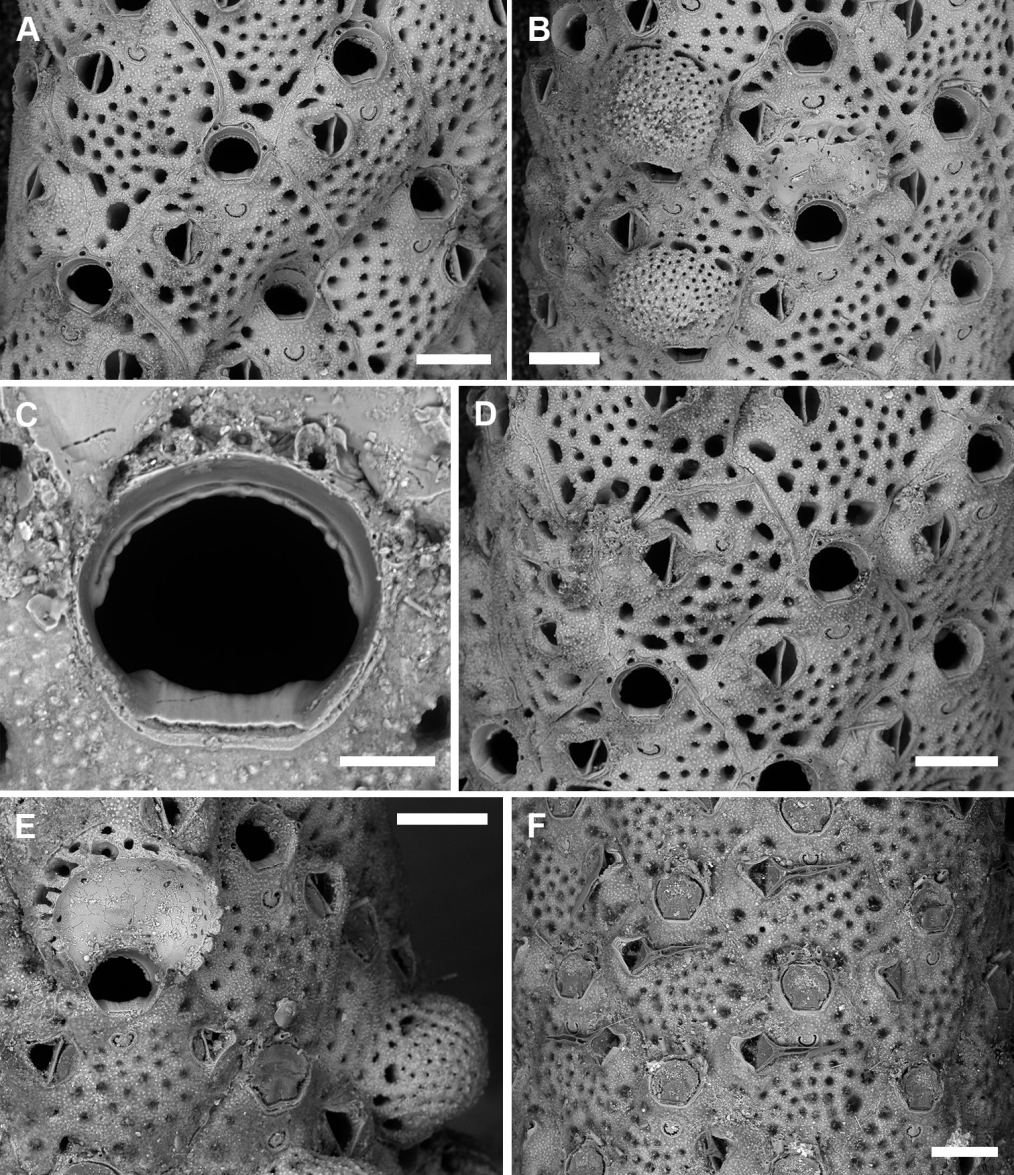
*Microporellaverrucosa* (Peach, 1868) from the Ionian Sea, Ciclopi Islands MPA, PMC. Rosso Collection I.H. B-17a **A** typical rhomboidal autozooids with large avicularia, transversely C-shaped fissure-like ascopore at the same level as the frontal shield, large pseudopores and larger marginal areolae, and thin oral spines surrounding the distal half of the orifice **B** autozooids, two of which have ovicells pierced by small, evenly distributed pseudopores, and outlined by a row of few, large, elongate pores **C** horseshoe-shaped orifice with blunt lateral condyles and slightly corrugated proximal border at the end of a smooth proximal shelf. Note the distal rim of calcification **D** a possibly abutted autozooid lacking the orifice **E** autozooids, one exceptionally developing paired avicularia **F** unbleached colony portion with opercula and open mandibles. Scale bars: 200 µm (**A, B, D–F**); 50 µm (**C**).

Autozooids rounded hexagonal to lozenge-shaped, 477–779 (661±93, N = 18) × 389–615 (493±68, N = 18) μm (mean L/W = 1.34), distinct, interzooidal boundaries marked by narrow, shallow, locally undulate grooves (Fig. 11A, B, D). Frontal shield nearly flat, finely granular, and pseudoporous; about 10 marginal areolae distinguishable from frontal pseudopores only in early ontogeny because larger, subcircular to elongate elliptical, 25–65 μm long; 19–26 subcircular pseudopores, 20–30 μm in diameter, placed centrally on the frontal (Fig. 11A, B, D). Transverse section of the branch showing thick frontal and vertical walls, converging towards the centre of the branch, forming wedge-like polypide cavities (Fig. 10E, F).

Primary orifice approximately semi-circular to horseshoe-shaped, 130–151 (143±6, N = 10) × 145–177 (161±10, N = 10) μm (mean OL/OW = 0.89; mean ZL/OL = 4.63), outlined by a thin and smooth raised rim (Fig. 11C); hinge-line smooth, straight to slightly concave with two short, blunt lateral condyles and a smooth, proximal shelf sloping outwards; distal margin of the orifice corrugated because of a deeply placed, drawstring-like, arched rim seemingly functioning as support for the closed operculum. Oral spines four, occasionally five, thin (base diameter 15–20 μm), relatively short (60–90 µm), placed distally, often detached (Fig. 11A, B, D).

Ascopore field a narrow, reniform to U-shaped rim of smooth gymnocystal calcification, 50–60 × 57–80 µm, placed 65–80 μm below the orifice, same level as the orifice and the adjacent frontal shield; ascopore opening transversely C-shaped, 40–63 × 5–12 μm, with a massive, upside-down mushroom-shaped tongue projecting from distal edge with radial spines (Fig. 11A, B).

A single, constant, large avicularium, 121–156 (142±9, N = 20) × 119–139 (130±7, N = 20) μm (mean AvL/AvW = 1.09), located laterally, on either side, at about half zooidal length (Figs 10B, C, 11A, B, D, F); crossbar complete; rostrum short, rounded triangular, channelled, directed laterally or less often distolaterally and slightly upward. Mandible 142–273 μm long, with a pointed, hooked tip, toothed at the level of the rostrum tip, lining proximally to the ascopore when open (Fig. 11F).

Ovicell non-personate, subglobular, prominent, large, 250–327 (286±36, N = 4) × 384–430 (402±20, N = 4) μm (mean OvL/OvW = 0.71), formed by the distal autozooid, obscuring half of the zooidal orifice; calcification fabric similar to frontal shield but with larger and more prominent tubercles, and smaller (15–20 μm in diameter), more closely spaced pseudopores, seemingly radially aligned with rows separated by raised ridges; a discontinuous, peripheral row of larger pseudopores sometimes present (Figs 10C, 11B, E).

Ancestrula tatiform (Fig. 10A, B), oval (220 × 150 μm), gymnocyst concealed, cryptocyst smooth, narrowing distally; opesia oval (160 × 110 μm); only four, distal spines visible on the single ancestrula observed. Ancestrula budding two distolateral autozooids, and subsequently surrounded by two lateral and one proximal autozooids.

Older colony parts thickly calcified owing to secondary calcification progressively obliterating zooidal openings including orifices, ascopores and avicularia (Fig. 10D), making difficult the distinction between old autozooids and genuine kenozooids that probably also develop.

###### Remarks.

First assigned to *Eschara* (Peach 1868), *Escharaverrucosa* served as the type species of the genus *Diporula* Hincks, 1879 in which it has been included since then with the exception of Neviani (1896a, b). Characters used to distinguish *Diporula* from *Microporella* included the “dendroid zoarium with cylindrical branches” and the morphology of the orifice described as “expanded above, contracted below, and slightly constricted by lateral projections (horseshoe-shaped)” (Hincks 1880: 220; and similar description in Gautier 1962: 176). However, both characters seem feeble to justify the distinction between the two genera. At least nine species of *Microporella* possess erect colony-growth, starting with a more or less developed encrusting portion as does *Diporula*. Also the shape of the orifice in *Microporella* species is highly variable (Di Martino et al. 2020a), with several examples of species having orifices with the proximal margins narrower than the orifice maximum width [e.g., *Microporellacurta* Almeida, Souza, Mengola & Vieira, 2017 from Brazil, *Microporellafranklini* (Soule, Chaney & Morris, 2003) from California, the Mediterranean *Microporellagenisii* (Audouin & Savigny, 1826), *Microporellahastingsae* Harmelin, Ostrovsky, Cáceres-Chamizo & Sanner, 2011 from the Red Sea, and the Arctic *Microporellaklugei* Kukliński & Taylor, 2008].

Further differences between *Microporella* and *Diporula* were highlighted by Hayward and Ryland (1999: 292, 312), including interzooidal communications via basal pore-chambers in the former genus and multiporous septula in the latter, and the presence of pseudopores in the ovicells of *Diporula*. However, multiporous septula were observed, for example, in *Microporellaordo* (see Di Martino et al. 2020a: fig. 7D), and basal pore-chambers were observed in the encrusting portions of *M.verrucosa*, while pseudopores occur in the ooecium of many *Microporella* species including the type *M.ciliata* (see Kukliński and Taylor 2008: fig. 1c). A further presumed difference relates to the ooecium porosity, with *Diporula* reported as having a fully perforated endooecium and *Microporella* species usually described as having only pits in the endooecium (Harmelin et al. 2011: 2; Ostrovsky 2013: figs 2.43B–D, 2.44A). However, pores clearly perforate the endooecium also in *Microporella* as seen in broken ooecia of *M.ichnusae* sp. nov. (Fig. 6B)

Based on these observations, here we propose *Diporula* as junior synonym of *Microporella* and resurrect the combination *Microporellaverrucosa* first proposed by Neviani (1896a, b). Specimens of a second species of *Diporula*, *D.coronula* Ortmann, 1890 need re-examination. Based on the original description and illustration (Ortmann 1890: 39, pl. 3, fig. 7), this species has a lepralioid orifice with condyles at about one-third of the orifice length, a single avicularium with spathulate mandible, and up to two frontal foramina, characters reminiscent of other cheilostome genera such as, for example, *Poricella* Canu, 1904.

Specimens originally described as *Escharalunaris* Waters, 1878, from Pleistocene sediment of eastern Sicily and synonymised with *M.verrucosa* by Hincks (1880) need to be re-examined as well to confirm their conspecificity, but this is out of the scope of the present paper.

The rugose appearance observed by Peach (1868) and Hincks (1880), which inspired the species name, was not observed in our material, although secondary calcification is always very common in older parts of the colony. Intramural budding is rare and restricted to avicularia, while branch regeneration is common, apparently following breakage as indicated by broken autozooids with sharp edges. We also observed zooids with reverse polarity, sometimes budded from old stems with autozooids obliterated by secondary calcification. However, in these instances few whorls of autozooids usually develop from the regeneration surface, with only few tips appearing actively growing. Colony fragments longer than 2 cm can appear twisted, a morphology observed in some cyclostomes (Harmelin 1976) and other erect cheilostomes from the Gulf of Noto and the Ciclopi MPA area (Rosso 1989). This twisted branch morphology and the ability to regenerate after breakage might represent the adaptation of this species to colonize soft sediment bottoms. Basal, encrusting colony portions are relatively common in our samples and show that the ancestrulae settled on clasts ranging from a few mm to 1–2 cm in size. The ability of this species to encrust small particles, in addition to large substrates in rocky habitats, was suggested by Gautier (1962) after finding only colony fragments in dredges from sandy-muddy bottoms.

The diagnostic characters of this species seem constant in the Mediterranean specimens, except for the size of the ascopore related to the development of the distal tongue sometimes leaving only a fissure-like opening. Paired avicularia were observed only in one autozooid (Fig. 11E). Higher variability is observed when comparing the Mediterranean specimens with those from the Atlantic (e.g., Hayward and Ryland 1999: 302, figs 138C, D, 139A, B; unpublished SEM images provided by P.D. Taylor from Mauritania and Madeira) related to the ascopore shape, the size of the spines, and the distribution and size of pseudopores on the frontal shield, suggesting the existence of a species complex.

###### Distribution and ecology.

*Microporellaverrucosa* is a warm-temperate species with Atlanto-Mediterranean distribution. In the Atlantic, it has been reported from West Africa to the southwest of the British Isles (Hayward and Ryland 1999); in the Mediterranean, it occurs preferentially in mid- and outer-shelf habitats below 50–60 m depth, with an optimum at 70–120 m (Gautier 1962; Zabala 1986; Rosso 1989, 1996a; Rosso and Di Martino 2016), but it was also observed at shallower depths (20 m) in a shadowed open cave in Catalonia (André et al. 2014). It is associated with shadowed rocky habitats, including the Coralligenous and Semi-Dark and Dark Cave Biocoenoses, and detritic habitats, such as the Coastal Detritic and the Offshore Detritic Biocoenoses (Table 1; Gautier 1962; Harmelin 1969, 1976; Zabala 1986; Rosso 1989, 1996a, 1996b; Di Geronimo et al. 1990; Madurell et al. 2013; Rosso et al. 2014, 2019b; Gerovasileiou and Rosso 2016). However, it is never very common or dominant at sample or habitat scale, occurring only with a few colonies per sample and/or in one out of four or five sampling stations (Table 1; see also Harmelin 1976: tables 1, 3).

**Table 1.** *Microporella* species studied in this paper with related sampling information and number of specimens found in each sample; numbers in brackets refer to dead specimens. Abbreviations for samples: PS/81, Piattaforma Siciliana cruise; N/96, Noto cruise; MZ and GR, Mazzere and Granchi caves from Plemmirio MPA; Cast. beach., Castelluccio beached; LCT, Gulf of Catania cruise; CI, Ciclopi 2000 cruise; AC and SM, Ciclopi Islands MPA samples; AM, Amendolara Bank cruise on board of Urania; PC, Porto Cesareo; Ta I, Taccio I; Ma, Madonna; Sc, Scoglio di Fora; ECE and EBE, Marettimo; CL, Calvì cruise of the University of Nice; AV, Agios Vasilios; c., cave; cn., Canyon; Isl., Island. Abbreviations for Biocoenoses: DC, Coastal Detritic Bottoms; DL, Offshore Detritic Bottoms; C, Coralligenous; VTC, Terrigenous Muddy Bottoms; DE, Muddy Detritic Bottoms; GSO, Semi-Obscure caves; IA, Infralittoral Algae; HP, Posidonia Meadow; GO, Obscure Caves; CB, Cold-Water Corals; na, not applicable; f, fragment.

Sea/ Locality	Sample	Depth	Biocoenosis	* appendiculata *	*bicollaris* sp. nov.	* ciliata *	*ichnusae* sp. nov.	* modesta *	*pachyspina* sp. nov.	sp. A	* verrucosa *
Ionian/ Gulf of Noto	PS/81 CR1	45	DC	27(8)				16(12)			
	PS/81 2B	65	DC								(2)
	PS/81 2C	83–74	DC								1(44)
	PS/81 2XA	128	DL								(4)
	PS/81 2XB	120	DL					(1)			(28)
	PS/81 4C	95–86	DL					(1)			(32)
	PS/81 4C1	89–84	DL								(44)
	PS/81 4X	102–93	DL					(1)			1(33)
	PS/81 6D	98–96	DL								(87)
	PS/81 9B	44	DC	(1)				(1)			
	PS/81 9C	60	DC					(1)			(3)
	PS/81 9D	78	DC					(12)			10(24)
	PS/81 10C	60	DC	(1)				(1)			(1)
	PS/81 11E	98	DL					(2)			(13)
	N/96 3C	20	C					11			
	N/96 5E	40	C-DC					1			
	N/96 6C	45	VTC	2							
	N/96 7E	35	C					12			
	N/96 8I	77	DE								1
	N/96 9E	50	DC	1							
	N/96 10G	82	DE					1			6
	N/96 10H	80	DE-DL								4
	N/96 10 I	107	DL					(1)			
	N/96 WP	90	DL			(1)		(24)			
Ionian/ Plemmirio caves	MZ1	≈23	GSO		(1)						
	GR E	≈19	C					3(1)			
Ionian/ Gulf of Catania	Cast. beac.	0	na					15			
	LCT69	90	DL					(2)			(18)
	Ognina	4	plastic					2			
	CI 2G	87.5	DE-DL					(4)			1(3)
	CI 3H	71	DC								(1)
	CI 4E	52	DC	1							(1)
	CI 6H	75	DC?								1(2)
	CI 8F	79	DC								(1)
	CI 8H	92.5	DE-DL								(1)
	CI 8I	95	DE-DL					(3)			(1)
	CI 9G	63	DC					(10)			(30)f
	CI 10G	85	DC-DE								(1)
	CI 12E	62	DC					(7)			(2)
	CI 12F	70	DC					(1)			(2)
	CI 12G	83	DE-DL					(5)			(1)
	CI 13H	105	DL								10(32) f
	CI 14G	90	DL	1							(1)
	AC/L	50	C								(16)
	AC/1B	110	DL								11(18) f
	SM1Z25	25	IA					1			
Ionian	Messina Strait	65	no data								2
Ionian/ Gulf of Taranto	AM 1D	30–40	DC					1			(3)
	AM 5D	40	DC					1			(1)
	PCI 10	5–15	C		2(1)						
Sicily Strait/ Pelagian Island	Ta I cave	10–20	C-GSO					1(1)			
	Ma cave	15	GSO					1			
	Sc cave	10	GSO					2(2)			
Sicily Strait/ Egadi Island	ECE 5	8	IA; IA-HP					ca.100	3		
	EBE/EBI	19							15		
Tyrrhenian	Palinuro c.	46	GO							(1)	
	Ustica Isl.	60	C					6(2)			(77)
Iberian-Provençal basin/Sardinia, Capo Caccia, and Asinara	Falco 1	7	GSO	2							
	Falco 2	4					3				
	Bisbe 1	8		2			1				
	Bisbe 2	8		1			4				
	Galatea 1	8					1				
	Galatea 2	6					1				
	PSE/PSI	5–15	IA					7			
W Corsica	CL 74	150–110	DL	(11)							(50)
Adriatic	Bari cn. 1B1	280	CB					(1)			
Aegean/ Lesvos Island	AV1	30	GSO	(1)							(1)
	AV2	30	GO								(3)

## Discussion

The present study increases the number of *Microporella* species known from the Mediterranean from nine (Rosso and Di Martino 2016; Di Martino et al. 2020a) to 14 (see Table 2 for a summary of species characters). The presence of *M.orientalis* and *M.coronata* remains based only on the records of Hayward (1974) from Chios and Harmelin et al. (2011) from Lebanon. Based on the description and illustrations of the ovicell as globose but non-personate (e.g., Zabala 1986: 513, fig. 180), further records of these two species are more likely to correspond to *M.appendiculata*. *Microporellamarsupiata*, although listed in Rosso and Di Martino (2016), can be considered restricted to the Atlantic. Mediterranean specimens identified as *M.marsupiata* (Zabala 1986) belong to *M.appendiculata*. This also applies to some specimens in our collection.

**Table 2.** Main diagnostic characters of Mediterranean *Microporella* species. Abbreviations are as follows. ; Colony form: E, encrusting; EC, erect cylindrical; EB, erect bilaminar. Ascopore: C, C-shaped; O, circular lacking distal denticle; R, reticulate. Ovicell: NP, non-personate; Pe, personate, ascopore not included; PA, personate, ascopore included; RI, visor-like proximal rim; ov, peristome arching on the ascopore. Pseudopores: ev perf, evenly perforate; centr, centrally; margin, marginally. Others: P, present; n.o., not observed; NO, not occurring. Numbers in brackets indicate formulas that are observed less commonly.

Species	Colony form	Ascopore	Frontal mucro	Avicularia	Ovicell	Pseudopores		Areolae	Oral spines		Orifice margin		Kenozooids
						Ovicell	Frontal		not ovicellate	ovicellate	Proximal	Distal	
* appendiculata *	E	O	P	2	NP; RI	absent	30–42	barely visible	5(6) first pair tri- or bifid	2	smooth	smooth	
*bicollaris* sp. nov.	E	C		1	PA; RI	1 row margin	11–25	3–6	5(6)	2	condyles at corners	smooth	
* browni *	E	C		1	Pe; RI	ev perf	31–98	barely visible	4–5 (3–7)	hidden	condyles, corrugated	beaded	
* ciliata *	E	C		1	NP	margin	30, central	4–5	1–4(0)	2	condyles laterally	smooth	
* coronata *	E	C	?	2	PA; RI	ev perf	50–60	1–3	7 (6–8)	2	condyles at corners	smooth	
* genisii *	E	C		1	Pe, ov; RI	less centr	18–30	barely visible	4 (3–6)	1–2	beaded	smooth	
*ichnusae* sp. nov.	E	C		1 (0–2)	NP	margin	10–30	2–4	4 (5–6)	hidden	condyles laterally	smooth	P
* harmeri *	E	C		1	P; RI	distal	45–90	5–9	3 (2–5)	hidden	11–20 teeth	smooth	
* modesta *	E, EB	C	P	1	NP	margin	50	6	5 (6–7)	2	condyles laterally	smooth	
* orientalis *	E	C		1	Pe	ev perf	?	?	0–5	hidden	condyles	beaded	
*pachyspina* sp. nov.	E	C	P	1	NP	1 row	6–18	3–6	5 (6–8)	2	condyles at corners	smooth	
sp. A	E	R		1	n.o.	n.o.	20–30	4–6	4–5	n.o.	condyles laterally	smooth	
* verrucosa *	EC	C		1 (0–2)	NP	small, large margin	19–26	10	4(5)	NO	sloping shelf; low condyles	corrugated	P

Modifications to genus and species diversity in the Mediterranean include the resurrection of the combinations *M.verrucosa* and *M.joannae*, and three new species described here (i.e., *M.bicollaris* sp. nov., *M.ichnusae* sp. nov. and *M.pachyspina* sp. nov.), as well as a potential additional species left in open nomenclature as *Microporella* sp. A. This is the result either of the re-examination of previously studied material assigned to the *M.ciliata* species complex or the examination of new material. *Microporellabicollaris* sp. nov. is clearly distinct from *M.ciliata*, given the presence of a personate ovicell. *Microporellaichnusae* sp. nov., *M.pachyspina* sp. nov., and the recently described *M.modesta*, on the other hand, have major affinities and share a certain number of features with *M.ciliata*. Furthermore, these species show high intracolonial and intraspecific variability common to other *Microporella* species and groups of species (e.g., Harmelin et al. 2011).

Most often, variability concerns the number of oral spines (e.g., 4–6 in *M.ichnusae* sp. nov.), 5–7 in *M.modesta*, 5–8 in *M.pachyspina* sp. nov., and 0–4 in *M.ciliata*), and the development of a tubercle on ovicells with the co-occurrence of tuberculate and non-tuberculate ooecia as in *M.modesta*. In this latter species, the mucro associated with the ascopore, considered as a diagnostic character by Di Martino et al. (2020a), also varies remarkably in the encrusting colonies examined here. In contrast, the size, density and distribution pattern of pseudopores on both autozooids and ovicells, as well as characters of the orifice, including the shape and size of condyles and denticulation of the hinge-line, are more stable. Harmelin et al. (2011) considered the variability of the denticulation of the orifice hinge-line in *M.harmeri* Hayward, 1988 (associated with differences in the morphology of the ascopore and in the shape of the avicularium and its mandible) as the indication that this widespread species, actually corresponds to a species complex. Periancestrular autozooids can lack avicularia in some species (e.g., *M.luellae* Grischenko, Dick & Mawatari, 2007, see Grischenko et al. 2007: fig. 34F), but the number of avicularia also seems constant within species, although sporadically autozooids may lack avicularia (as in *M.ichnusae* sp. nov.) or bear a second avicularium (as in *M.ichnusae* sp. nov. and *M.verrucosa*). Paired avicularia were also described in “*M.ciliata*” by Zabala (1986) and appear in *Microporellaketchikanensis* Dick, Grischenko & Mawatari, 2005 (Dick et al. 2005: fig. 20F) and *M.stellata* (Winston et al. 2000: fig. 19). Autozooids lacking avicularia have been occasionally reported in *Microporella* species, such as the Pliocene *Microporellasarasotaensis* Di Martino, Taylor & Portell, 2019 from Florida (Di Martino et al. 2019: fig. 37), the Pleistocene *M.rusti* from New Zealand (Di Martino et al. 2017: fig. 4), and the present-day *M.maldiviensis* Harmelin, Ostrovsky, Cáceres-Chamizo & Sanner, 2011 from the Maldive Islands (Harmelin et al. 2011: fig. 12I), and *M.ketchikanensis* (Dick et al. 2005: fig. 20A). The three latter species also show autozooids developing paired avicularia, thus paralleling *M.ichnusae* sp. nov. for the co-occurrence of autozooids with 0, 1, or 2 avicularia.

Kenozooids, with or without an avicularium, are here mentioned for the first time for Mediterranean species. Kenozooids were observed in *M.ichnusae* sp. nov., *M.modesta* and *M.verrucosa*, often along the contact zone between two different colonies or lobes of the same colony, as already seen in fossil species of *Microporella* involved in competitive interactions (Di Martino et al. 2020b).

Of the Mediterranean *Microporella* species examined here, *M.verrucosa* and *M.modesta* are the most frequently recorded (Fig. 1). *Microporellaverrucosa* is also the only Mediterranean erect species with cylindrical branches forming relatively large colonies, therefore easily recognisable. The identical zooidal morphology of erect species of *Microporella* (*M.hastigera*, see Di Martino et al. 2020a) with those originally assigned to *Diporula*, such as *M.verrucosa*, led to the suggested synonymy between these two genera. The main difference between the two genera related to the porosity of the ooecium does not hold (see also Remarks for *M.verrucosa*): *Microporella* species can have either evenly pseudoporous ooecia with pores penetrating the endooecium (e.g., *M.ichnusae* sp. nov., Fig. 6B), or ooecia with a single row of marginal pores imperforate centrally (e.g., *M.pachyspina* sp. nov., Fig. 8E), as well as imperforate ooecia (e.g., *M.appendiculata*, Fig. 2E). The lack of pseudopores in the ooecium is also a feature of the Langhian *M.berningi* Zagorsek, 2010 from the Czech Republic, which also shares with *M.appendiculata* the formation of ooecia by kenozooids that are therefore independent of the distal autozooids, which is likely the reason for the lack of peripheral areolar pores (Fig. 2C, D). Although based on a single species, “*M.ciliata*”, the ooecia in *Microporella* have been defined as microporelliform (i.e., an outgrowth of the proximal part of the frontal shield of the distal autozooid) (Ostrovsky 2013: 141, fig. 2.43A–D), which does, therefore, not apply to at least some of the species.

Another *Microporella*-like genus, *Flustramorpha* Gray, 1872, recorded off the coast of South Africa, is likely to be also indistinguishable from *Microporella*. However, species assigned to *Flustramorpha* need to be revised before the synonymy can be considered.

*Microporellamodesta* has high growth plasticity and, although first described as erect bifoliate, encrusting colonies are more common. No habitat information is available for the type locality off the coast of Algeria, while colonies studied here are from shelf habitats. At shallow depths, *M.modesta* preferentially lives as an epibiont of soft and ephemeral substrates such as seagrasses and algae, and also on geniculate corallines, while in deeper environments it is mainly associated with other bryozoans. Despite the large number of colonies examined, no evidence was found of bilaminar branches starting from the encrusting base. The revision of large bryozoan collections from the Mediterranean (i.e., Rosso’s collection, this study; Chimenz Gusso’s collection in Chimenz Gusso et al. 2014), has shown that those species, including *M.ciliata*, previously considered widespread in the Mediterranean, are instead restricted to some specific areas and habitats. For instance, *Microporellaichnusae* sp. nov. and *M.* sp. A were found only in submarine cave habitats, with the former species inhabiting relatively lit caves of NW Sardinia (E Iberian-Provençal basin) shallower than 10 m, and the latter species collected dead at 46 m depth in a completely dark cave of the Palinuro Peninsula (E Tyrrhenian Sea). *Microporellabicollaris* sp. nov. and *M.pachyspina* sp. nov. seem to be southern species, inhabiting shallow-water habitats from the Sicily Strait, mostly associated with the Infralittoral Algae and the *Posidonia* meadow Biocoenoses.

In summary, based on the above discussion, some characters were observed for the first time in *Microporella*: tatiform ancestrula regenerated as an autozooid or kenozooid; presence of kenozooids; interzooidal communication through basal pore chamber windows in encrusting species or encrusting base of erect species and multiporous septula in erect branches; ovicells of kenozooidal origin; erect species with cylindrical branches (owing to the displacement of *M.verrucosa*).

## Conclusions

With the description of three new species (i.e., *M.bicollaris* sp. nov., *M.ichnusae* sp. nov., *M.pachyspina* sp. nov.), and the inclusion of *M.verrucosa* and *M.joannae*, this study confirms *Microporella* as one of the most species-rich genera of the Mediterranean, after *Schizomavella* and *Reteporella*, with 22 and 15 known species, respectively (see Rosso and Di Martino 2016). Three or even four *Microporella* species, such as those recorded or described from the Mediterranean eastern sectors, are considered non-indigenous and possibly introduced in recent times, mostly via the Suez Canal (Harmelin et al. 2011) as suggested by the fact that they are all shallow-water species (3–43 m depth) found in harbours, marinas, and nearby natural environments. For the new species described here, their status as native or alien species cannot be assessed with certainty, although two of them, *M.bicollaris* sp. nov., *M.pachyspina* sp. nov., but also *M.* sp. A, show their highest affinities with extra-Mediterranean species, specifically with species from the Pacific Ocean, including the Galapagos Islands and the Arctic, areas that are geographically distant and characterised by decidedly different environmental parameters. An anthropogenically-mediated transport might be hypothesised for the first two species that have a shallow-water distribution, often in association with algal fronds. However, a similar widely disjunct distribution was observed for the calescharid species *Tretosinaarculifera* (Canu & Bassler, 1927), previously only known from deep-waters of Hawaii and recently discovered in submarine caves from Lesvos Island (Rosso et al. 2020b). To date, there is no evidence of the occurrence of *Microporella* species in highly human-impacted areas (Ferrario et al. 2018), with the only exception of colonies of *M.modesta* found on plastic at Ognina marina, north of Catania.

Like for some other genera (e.g., *Setosella* Hincks, 1877; see Rosso et al. 2020a), the description of such a significant number of new species shows how incomplete our knowledge of the bryozoan biodiversity in the Mediterranean basin still is, despite being one of the most investigated areas during the past few centuries (see Rosso and Di Martino 2016, and references therein).

## Supplementary Material

XML Treatment for Microporella

XML Treatment for Microporella appendiculata

XML Treatment for Microporella bicollaris

XML Treatment for Microporella ciliata

XML Treatment for Microporella ichnusae

XML Treatment for Microporella modesta

XML Treatment for Microporella pachyspina

XML Treatment for Microporella

XML Treatment for Microporella verrucosa
